# Metabolomics and Lipidomics: Expanding the Molecular Landscape of Exercise Biology

**DOI:** 10.3390/metabo11030151

**Published:** 2021-03-07

**Authors:** Mehdi R. Belhaj, Nathan G. Lawler, Nolan J. Hoffman

**Affiliations:** 1 Exercise and Nutrition Research Program, Mary MacKillop Institute for Health Research, Australian Catholic University, Melbourne 3000, Australia; mehdi.belhaj@acu.edu.au; 2 Australian National Phenome Centre, Health Futures Institute, Murdoch University, Harry Perkins Building, Murdoch, Perth 6150, Australia; nathan.lawler@murdoch.edu.au; 3 School of Health and Medical Sciences, Edith Cowan University, Joondalup 6027, Australia

**Keywords:** exercise, metabolism, omics, metabolomics, metabolome, lipidomics, lipidome, mass spectrometry, nuclear magnetic resonance

## Abstract

Dynamic changes in circulating and tissue metabolites and lipids occur in response to exercise-induced cellular and whole-body energy demands to maintain metabolic homeostasis. The metabolome and lipidome in a given biological system provides a molecular snapshot of these rapid and complex metabolic perturbations. The application of metabolomics and lipidomics to map the metabolic responses to an acute bout of aerobic/endurance or resistance exercise has dramatically expanded over the past decade thanks to major analytical advancements, with most exercise-related studies to date focused on analyzing human biofluids and tissues. Experimental and analytical considerations, as well as complementary studies using animal model systems, are warranted to help overcome challenges associated with large human interindividual variability and decipher the breadth of molecular mechanisms underlying the metabolic health-promoting effects of exercise. In this review, we provide a guide for exercise researchers regarding analytical techniques and experimental workflows commonly used in metabolomics and lipidomics. Furthermore, we discuss advancements in human and mammalian exercise research utilizing metabolomic and lipidomic approaches in the last decade, as well as highlight key technical considerations and remaining knowledge gaps to continue expanding the molecular landscape of exercise biology.

## 1. Introduction

Living systems maintain metabolic homeostasis thanks to countless chemical reactions that continuously change the molecular landscape within these biological systems, including biofluids and tissues throughout the body. The term metabolism—derived from the Greek word “metabole” meaning “change”—defines all the chemical reactions that change molecules within living systems. Exercise represents a major challenge to whole-body and cellular energy homeostasis, and a multitude of molecular responses to acute exercise (i.e., a single exercise bout) are engaged to combat energy stress at the cellular and whole-body level [1]. During an intense acute exercise bout, the cellular turnover of adenosine triphosphate (ATP)—the energy “currency” of the cell—can increase 100-fold relative to the resting state, while at the whole-body level, the metabolic rate can increase up to 20-fold [2]. Given the small concentrations of readily available ATP in skeletal muscle cells (~8 mmol/kg wet weight) [3], ATP-resynthesizing pathways are rapidly activated in response to exercise to help maintain ATP concentrations within the working muscle and facilitate muscle contraction [4]. These cellular pathways responsible for ATP generation include: (1) the ATP-phosphocreatine (ATP-PCr) system whereby the breakdown of PCr produces free Cr and inorganic phosphate (P_i_) that is subsequently transferred to ADP to resynthesize ATP; (2) “anaerobic” glycolysis where glucose units mainly derived from intramuscular glycogen and circulating glucose are catabolized to pyruvate and reduced to lactate, generating ATP by substrate phosphorylation; and (3) carbohydrate and lipid breakdown (glycolysis and β-oxidation, respectively) producing acetyl-CoA which subsequently enters the tricarboxylic acid (TCA) cycle in the mitochondria and produces electrons that are transferred through the electron transport chain, resulting in ATP generation [5]. The relative contribution of these different pathways and the related substrates utilized to help fuel working skeletal muscle are mainly dictated by exercise intensity and duration [2,6,7,8]. The metabolic perturbations provoked by exercise are however not restricted to working muscles and engage numerous cell types and organs throughout the body to help meet the metabolic demands of exercise [1].

Although tremendous progress in the understanding of the cellular and molecular mechanisms involved in the responses to acute exercise has occurred over the past decades through traditional “reductionist” approaches, these approaches are limited to studying a biochemical pathway or molecular target of interest in isolation. As a result, further insight into the complex integrative nature of exercise-regulated molecular metabolic networks has been limited, and critical knowledge gaps remain [1,9]. Fortunately, the growing application of global “omics” approaches to unravel metabolite and lipid network responses to exercise in the last decade has marked an important turning point in this research area. These omics-based approaches have introduced new opportunities to better decipher the complexity and interconnection of exercise molecular transducers and their potential contributions to exercise’s wide range of health benefits. In this review, we introduce these omics-based approaches to exercise researchers and provide a critical overview of the last decade of metabolomic and lipidomic applications, two of the most recently introduced omics-based approaches, to studying the molecular responses to acute exercise in humans and other mammalian species. Furthermore, we discuss key technical considerations, remaining knowledge gaps and hurdles associated with metabolomics and lipidomics, as well as highlight future research directions to continue expanding the molecular landscape of exercise biology.

## 2. Metabolomics and Lipidomics Guide for Exercise Researchers

### 2.1. What Are Metabolomics and Lipidomics?

Metabolomics is defined as the comprehensive study of metabolites present in a given biological system (e.g., biofluid, tissue) [10,11]. The metabolome—a term first coined by Oliver and colleagues in 1998—represents the entire collection of metabolites within a biological system [12]. Metabolites are defined as low molecular weight (<1500 Daltons) chemical substrates, intermediates or end products of enzyme-mediated reactions [10]. The study of the metabolome is also commonly referred to as “metabonomics”, which was first defined as “the quantitative measurement of the dynamic multiparametric metabolic response of living systems to pathophysiological stimuli or genetic modification” [13]. This definition was later expanded to include the “particular emphasis on the elucidation of differences in population groups due to genetic modification, disease, and environmental (including nutritional) stress” [14]. Although differently defined, the terms metabolomics and metabonomics are often used interchangeably [15].

Metabolites are heterogeneous compounds that possess various physicochemical properties, but are generally classified as either hydrophilic polar molecules (e.g., amino acids, carbohydrates, organic acids and phosphorylated compounds) or hydrophobic non-polar molecules (e.g., fatty acids and membrane lipids) [16]. The human metabolome is comprised of thousands of metabolites, with the latest report from the Human Metabolome Database (HMDB) in December 2020 indicating no less than 8000 endogenous metabolites detected [17], while almost 35,000 exogenous metabolites from sources such as foods, drugs, toxins and microbes have been detected and/or expected [18].

Lipidomics, considered a subfield of metabolomics [16,19], is the study of the lipidome, i.e., the total lipid content within a cell, organ or biological system [20]. Lipids are often simply defined as hydrophobic biological substances generally soluble in organic solvents [21]. However, lipids can be more accurately characterized based on structural or biosynthetic criteria [22]. The LIPID MAPS^®^ consortium has provided a lipid classification system including a comprehensive list of lipid categories (e.g., fatty acyls, glycerolipids, sphingolipids, sterol lipids), classes and subclasses [22]. As of December 2020, the LIPID MAPS^®^ Structure Database contains more than 45,000 unique lipid structures [23].

Emerging only at the start of the new millennium, metabolomics and lipidomics represent the newest applications among global omics-based approaches (i.e., genomics, transcriptomics, proteomics, phosphoproteomics). The rapidly growing fields of metabolomics and lipidomics have dramatically expanded over the past 15–20 years, thanks to major advancements in analytical instrument technologies and bioinformatic analysis platforms. As a result, there has been a substantial increase in the application of metabolomics and lipidomics across a wide range of research fields, including health and disease [24], toxicology [25], nutrition [26] and exercise physiology [27]. The growing application of these omics-based technologies therefore represents a promising avenue to continue improving our understanding of the complexity and interconnection of exercise-regulated metabolic reactions within biological systems, which were previously limited by the application of traditional reductionist approaches only studying targeted metabolite(s) of interest in isolation [10].

### 2.2. Advantages to Studying the Metabolome and Lipidome in Biological Systems

**Figure 1:**
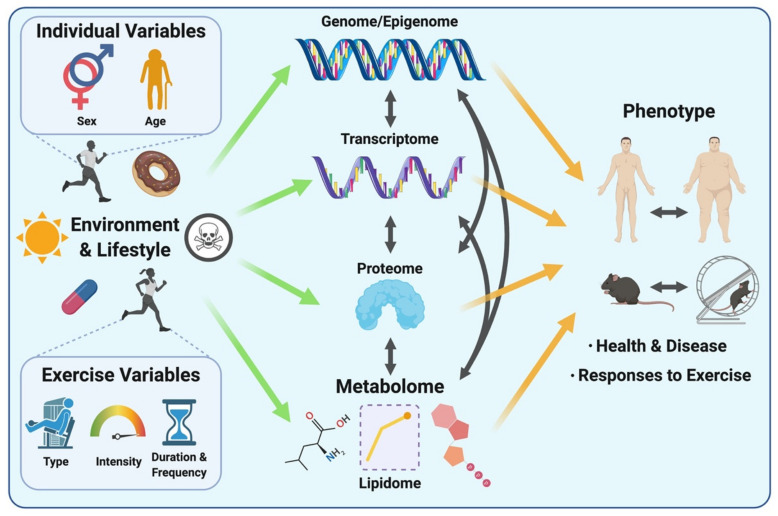
The complex interrelations between biological layers (from the genome/epigenome, transcriptome and proteome to the metabolome) and other individual factors (sex and age, environment and lifestyle including exposure to toxins and pollutants (symbolized by skull and crossbones), medication use, dietary habits, …) and exercise variables, and how these biological networks and variables contribute to the overall phenotype. Environmental exposures and lifestyle, including diet and medication, as well as exercise and its associated variables (exercise type, intensity, duration and frequency) can affect all layers of biological regulation and lead to distinct phenotypic signatures in mammalian systems that reflect health, disease and responses to exercise. Adapted from [10].

Studying the metabolome (including the lipidome) is appealing for expanding our understanding of complex biological systems in the context of exercise, as metabolites lie downstream of all other layers of biological regulation. Therefore, the metabolome reflects the cumulative changes resulting from processes involving the genome, transcriptome and the proteome, as well as their interactions with the environment (Figure 1). The metabolome thus directly reflects the phenotype of a given biological system at the molecular metabolic level [10]. Put differently, while genomics, transcriptomics and proteomics altogether provide a program of what might occur within a biological system, metabolomics provides a snapshot of phenotypic traits (i.e., phenome), revealing what is currently occurring or has happened as a result of these other layers of biological regulation [28,29]. Considering rapid metabolite turnover, which can be detected in seconds versus minutes to hours for transcriptomic and proteomic responses to a stimulus such as an acute exercise bout, the metabolome serves as a rapid indicator of metabolic perturbations and chemical reactions occurring as a result of post-translational regulation (e.g., protein phosphorylation) in response to a given stimulus or environmental exposure.

Another advantage of studying the metabolome/lipidome is that the central reactions and pathways essential to energy metabolism, growth and nutrient supply are highly conserved across mammalian species, meaning that metabolite measurements obtained from other mammalian species such as rodents may be relevant and translational to humans [10,30]. In addition, the transferability of analytical methods across distinct biological systems (e.g., biofluids, tissues) makes metabolomic/lipidomic approaches attractive by dramatically reducing labour and time associated with optimization, and providing high-throughput data at relatively low cost per sample compared with other omics-based approaches such as transcriptomics and proteomics [10]. Another advantage is the small sample volumes (typically 10–100 µL) required for compound detection, identification and quantification, which in turn makes collection of multiple samples in relatively short periods of time feasible (e.g., serial blood sampling at close intervals during/after exercise). Finally, when using an untargeted approach as detailed below, metabolomics/lipidomics permits the detection of changes in previously unknown, uncharacterized or rarely reported metabolites [31]. This approach allows for potential hypothesis generation and can facilitate retrospective data analysis to unlock potential mechanisms linked to disease or intervention strategy.

### 2.3. Types of Metabolomic and Lipidomic Approaches

Omics-based approaches such as metabolomics and lipidomics are generally described as unbiased, global experimental strategies to identify and/or quantify as many compounds as possible within a biological system. However, different approaches to investigating metabolites and/or lipids within a biological system are currently available, as briefly outlined below and reviewed elsewhere in more detail [10,32]:

#### 2.3.1. Untargeted Approach 

This approach aims to reproducibly measure as many metabolites/lipids as possible in a given biological sample. Depending on the platform utilized, the untargeted strategy generally yields a metabolite detection coverage in the hundreds to low thousands using a combination of different separation and detection modes (described below). This approach provides semi-quantitative data, meaning that peak areas are reported for each metabolite instead of absolute concentrations. These peak areas allow the assessment of the relative abundance of detected metabolites between experimental groups. Of note, metabolite identities are usually unknown prior to data acquisition and analysis when using this approach. 

#### 2.3.2. Targeted Approach

As opposed to the untargeted strategy, the targeted approach aims to provide absolute concentrations of a set of known metabolites (ranging from one to 100 metabolites, typically a few dozen, depending on the number of compounds of interest) by using authentic chemical standards and calibration curves for each pre-selected metabolite. Recent developments in commercially available targeted metabolomics kits now facilitate the identification of up to 188 metabolites, and up to 1184 different lipids for lipidomics [19].

#### 2.3.3. Semi-Targeted Approach 

This third approach is less common. However, it is gaining popularity amongst many metabolomics research groups. This approach represents an intermediate strategy between untargeted and targeted approaches where a higher number of known metabolites (i.e., low hundreds) are investigated using a single chemical standard and ad hoc calibration curve for multiple metabolites, thus providing approximate metabolite concentrations.

The untargeted approach is primarily used as a hypothesis-generating method where the research question is generally unbiased with respect to metabolite identification. In contrast, the targeted approach can be used as a hypothesis-testing and experimental validation method, as it provides much higher sensitivity and specificity in comparison to the untargeted approach, but with reduced metabolite coverage. Therefore, the untargeted approach permits the identification of new potential biomarkers and pathways, which can be further validated and more accurately quantified via the targeted approach. Although these approaches are complementary, the targeted strategy provides the most quantitative insights into metabolite dynamics in response to stimuli such as exercise [16].

### 2.4. Commonly Used Metabolomic and Lipidomic Analytical Platforms

Regardless of the type of metabolomic approach utilized, the main analytical platforms used in metabolomic studies are mass spectrometry (MS) and nuclear magnetic resonance (NMR) spectroscopy [16,19]. We provide a brief overview of NMR spectroscopy and MS principles below, and readers are referred to the following review articles for further details of these analytical techniques [33,34,35,36]. 

Briefly, NMR spectroscopy is a technique based on the interaction of an applied magnetic field with the nuclei of atoms which possess an odd number of protons or neutrons, including ^1^H, ^13^C, ^15^N, conferring their magnetic properties. The magnetic orientations of these atoms, which have random directions, become aligned when a magnetic field is applied. Then, a pulse of electromagnetic radiation at a specific “resonance” frequency (dependent on the atom and magnetic field) is applied, causing nuclei “excitation” and subsequent “relaxation” when the radiation pulse stops. During relaxation, nuclei emit the radiofrequency waves absorbed during the excitation phase, thus generating radiofrequency peaks in a frequency spectrum (also called NMR spectrum) after Fourier’s transformation. 

NMR spectroscopy is used as a non-destructive technique and allows the measurement of chemical and physical properties of molecules, therefore helping identify and/or quantify molecules of interest. In theory, this can be performed in solid, liquid and gas states from frozen to very high temperature materials, although researchers typically focus on only one aggregation state based on practicality and feasibility. Numerous applications using “pulse sequence” have been developed to expand the capability of NMR techniques. Pulse sequence is analogous to music scores for an orchestra to create the right harmony, which is the spectrum in NMR. Application of the right pulse sequence can enable sample information such as chemical structure, molecular shape, size and molecular aggregation to be obtained. Since NMR is non-destructive, the sample can be reused to gain more information using different pulse sequences, unless the sample condition has changed during the experiment (e.g., heat application, temperature change). The major limitation of the NMR technique is low sensitivity compared to MS, resulting in reduced metabolite coverage (typically 50–200 metabolites detected and identified) with NMR [37,38]. Compared to MS, NMR also requires more sample volume (~0.28–0.5 mL) to obtain measurements. However, advantages of NMR over MS include the ability to analyze living samples (e.g., magnetic resonance imaging) and the ease in distinguishing compounds of identical molecular weight with NMR [38].

In MS-based techniques, the compounds present in a biological sample are converted to ions (with either a positive or negative charge) through the use of an ion source. The formed ions then enter the MS instrument which detects ions and their abundance, providing a mass spectrum displaying mass-to-charge ratio (*m*/*z*) and peak intensity (i.e., ion abundance). Further information can be collected through tandem MS (MS/MS or MS^2^) in which either intact “precursor” ions are fragmented into “product” ions, or ions already fragmented during MS undergo further fragmentation, providing additional structural information about a given compound detected and thus enhancing compound identification capacity. MS^2^ is particularly useful for compounds with identical *m*/*z* values [34,36]. 

Although MS can be used as standalone instrumentation for metabolite detection and identification, i.e., direct infusion MS (DIMS), it is typically but not exclusively combined with chromatographic separation techniques such as gas chromatography (GC) and liquid chromatography (LC). Chromatography columns contain a stationary phase that interacts with the sample and uses the affinity of molecules to separate them within complex matrices. As molecules flow along the column, their different affinities for the stationary phase result in different retention times in the column and thus sequential introduction into the MS instrument, therefore enhancing sensitivity and identification capacity [34,37]. Due to major technological advances over the past two decades, LC-MS is currently the most widely used technique in metabolic profiling. Indeed, LC-MS provides high metabolite coverage, reproducibility, specificity and sensitivity [37]. Similar to LC-MS, GC-MS has a strong capacity for separation, sensitivity, selectivity and reproducibility. However, GC-MS can only be used for the separation and identification of volatile compounds and low molecular weight compounds (50–600 Da) [39], and such chemicals must be volatile or amenable to chemical derivatization to render them volatile. Each analytical platform has its own advantages and limitations and should therefore be considered complementary rather than opposing analytical techniques to provide comprehensive metabolomic analyses. No single platform can yield detection, identification and quantification of the full range of metabolites within a given biological sample and as such, multiple separation techniques and analytical platforms may be used in combination to increase metabolite coverage [19,37]. More detailed information about specific methods, advantages and limitations regarding the use of GC-MS, LC-MS (including MS/MS) and NMR for metabolomics have been extensively reviewed elsewhere [37,38,39].

Lipidomics shares similar analytical techniques with metabolomics [20,40]. Although NMR spectroscopy is considered a powerful tool for lipid identification [41], the lipidomics field has predominantly applied MS-based techniques with numerous potential experimental and sample preparation variations. The most commonly used MS techniques can be divided into two categories: (1) direct analysis from a biological matrix; and (2) analysis following lipid extraction, with or without subsequent separation [20,40]. 

Direct analysis from biological matrices is mainly performed by MS imaging. An appealing characteristic of this method is its ability to determine the spatial distribution of thousands of lipid species in tissue sections without any labelling [42]. The principle of MS imaging is similar to classical MS in that compounds from the sample (i.e., tissue section) are ionized, for example using matrix-assisted laser desorption ionization (MALDI) or desorption electrospray ionization (DESI) and analyzed by MS. The main distinction between MS imaging and classical MS is that a tissue section is divided into squares or pixels with MS imaging, and compounds within each pixel are subject to ionization and MS, pixel by pixel. Mass spectra are acquired for each pixel and specific *m*/*z* values can be individually selected to visualize their signal intensity (thus abundance) within the tissue section. By merging the different colour-coded *m*/*z* signals, spatial distribution and abundance of different lipid species can be visualized throughout the tissue [43,44]. Another interesting characteristic of MS imaging is that this approach requires minimal sample preparation (with MALDI-MS imaging) or no preparation (DESI-MS imaging) other than tissue sectioning [20].

The analysis of lipid extracts without separation is often referred to as “shotgun” lipidomics or direct infusion-based lipidomics, whereby a given lipid extract is continuously injected in the MS instrument, generally after selective ionization by an ion source, which provides some lipid separation [20]. Despite various advantages, limitations associated with shotgun lipidomics include ambiguous identification of lipid isomers and ion suppression [45]. Ion suppression is a phenomenon that results from the presence of less ionizable/volatile compounds which affects the efficiency of droplet formation or evaporation, thus leading to a reduction in charged ions in the gas phase that enter the mass spectrometer [46]. Most limitations of shotgun lipidomics are overcome by multidimensional MS-based shotgun lipidomics (MDMS-SL), which integrates a full mass scan (first dimension) and all MS/MS scans (second dimension) for head groups and acyl chains, thus allowing the identification of individual lipid species (including isomers) and providing accurate quantification but with relatively low throughput [20]. Lipid annotations are then based on accurate mass and fragmentation patterns, which is facilitated by reference databases [47,48]. In contrast, separation methods prior to MS analysis allow minimal ion suppression. Among these separation methods, LC-MS is the most widely used for these same reasons, among others, as detailed above [20,40,45]. Nestled between LC and MS is a very fast separation technique called ion mobility spectrometry (IMS), which is used to provide an additional dimension of separation based on ions’ shape and size, known as collisional cross section (CCS). This technique is becoming particularly important for lipids as it allows the separation of isomers using trapped ion mobility spectrometry (TIMS) and a gas flow which facilitates lipid identification [49].

### 2.5. Overall Metabolomic and Lipidomic Workflow

**Figure 2:**
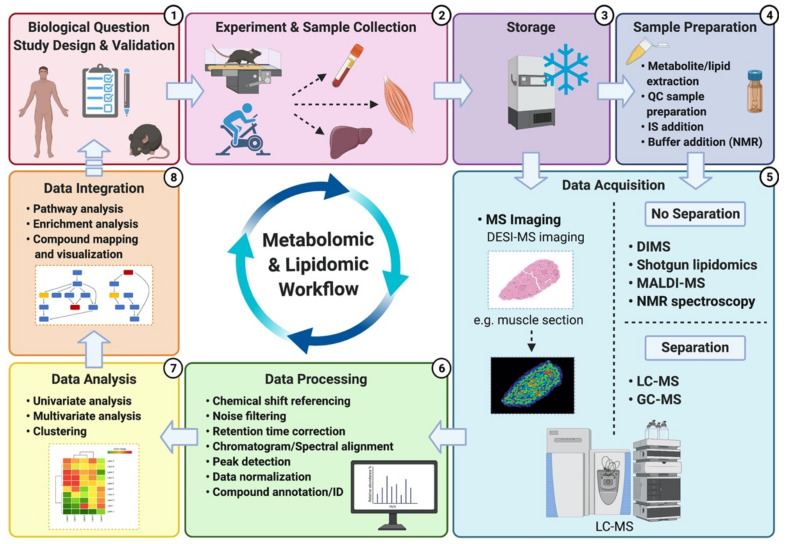
Typical metabolomics/lipidomics workflow: (**1**) After establishing a given biological question, appropriate and optimized study design is a critical step to answer this biological question with minimal bias and noise (i.e., investigation-induced variability). (**2**) Sample collection before/during/after the experiment also requires particular attention and care to avoid introducing potential biases. Therefore, consistency of collection timing, materials and reagents is important. Metabolic reactions are rapid and must be stopped as soon as possible following collection by snap freezing or placing the sample on ice. (**3**) Samples are then prepared accordingly (e.g., centrifugation of whole blood to collect plasma or serum) for storage until planned sample preparation or direct data acquisition. (**4**) Sample preparation depends on the analytical platforms utilized and the molecular species to be extracted (e.g., lipids or other metabolites). During this step, QC samples are usually prepared and IS added to all aliquots to screen and correct platform-related shifts and enhance reproducibility. (**5**) Samples are analyzed and data are acquired using one or multiple analytical platforms. (**6**) Acquired raw data are then processed through multiple steps to eventually allow accurate compound identification/annotation. (**7**) Multiple statistical tests are performed on the identified/annotated compounds to determine potential differences between samples and/or groups in line with the biological question and experimental design. (**8**) Finally, data are placed into biological context using pathway/enrichment analysis and visualization tools, which also help inform future biological research questions and experimental designs, therefore leading back to step one of the workflow. Alternatively, targeted validation of metabolites/lipids of interest within the dataset may be performed following data integration.

A typical untargeted metabolomics/lipidomics workflow is composed of several experimental and analysis steps (Figure 2). The first step of this workflow is establishing the experimental question and optimizing the study design. A robust study design is crucial to ensure minimal investigator-induced variation in the biological sample and subsequent reduction of noise within the metabolomic/lipidomic dataset, which can eventually hinder confidence of data interpretation [31,32]. The following step includes performing the experimentation and the resulting sample collection, storage and preparation. These steps are also critical since many biases may be introduced, potentially altering the metabolite/lipid composition of the biological sample [50]. Consistency of experimental methods (e.g., timing of collection, materials and reagents, storage temperature) is paramount to enable acquisition of accurate and reproducible results [36]. Sample preparation methods and reagent selection will mainly depend on the sample type (e.g., blood, urine, saliva), platforms being utilized (e.g., NMR versus MS), and compounds of interest (lipid classes versus all metabolites). Whereas NMR only requires minimal and non-sophisticated sample preparation [19], MS-based platforms can require additional preparative steps for the inclusion of quality control (QC) samples and internal standards (IS) for the generation of calibration curves and accuracy check. However, both instruments will typically use QC samples within the analysis to check for reproducibility and, for MS, to monitor/correct potential shifts in mass accuracy and retention times [31,32]. 

Next, data acquisition refers to the detection and characterization (e.g., *m*/*z* ratio and peak intensity in MS; and chemical shift in NMR, i.e., resonance frequency of a nucleus relative to a standard with a value of 0) of the compounds present in samples through the use of one (and sometimes several) of the analytical platforms mentioned above. Once acquired, raw peak intensity data are processed to permit further analysis. In MS, data processing comprises many steps including conversion of raw peaks into data matrices, noise filtering, retention time correction, chromatogram alignment, peak detection, data normalization, and eventually, compound “putative” identification by matching metabolite/lipid spectra against in-house libraries and available databases such as HMDB, METLIN or LIPID MAPS. Putatively identified compounds are then benchmarked by the investigator, and the relative levels of identification confidence are assigned and reported according to the Metabolomics Standard Initiative (MSI). The (MSI), and more recently, the Lipidomics Standards Initiative (LSI), have notably been created to standardize the confidence levels for metabolite and lipid identification [41,51]. In NMR, different steps precede compound identification/annotation including: spectral pre-processing consisting of noise reduction and baseline correction; sub-spectral selection where only areas of the spectra containing peaks are kept; spectral alignment; spectra division into sections (i.e., bins) that can fit one or more peaks; followed by calculation of bin intensities and statistical tests to assign bins to a specific metabolite. Data normalization, scaling and transformation are also performed prior to data analysis and interpretation [52]. 

Following identification (regardless of the analytical platform used), a broad range of statistical analyses is performed to determine potential differences between samples and/or experimental groups. Commonly used statistical methods include univariate and multivariate analysis, either in an unsupervised or supervised manner. ANOVA and *t*-test or nonparametric equivalents are widely used univariate analysis methods, whereas principal component analysis (PCA) and partial least square-discriminant analysis (PLS-DA) are common examples of unsupervised and supervised multivariate methods, respectively [53]. Briefly, the use of PCA can reveal patterns or signatures within the sample set and show sample reproducibility through clustering of quality control samples within and between batches. PLS-DA is a predictive and descriptive modelling technique used for classification between different groups of samples and optimizes separation between these groups of samples [53,54]. A plethora of statistical methods are available, but there is no one size fits all approach. The choice of suitable statistical methods will depend on the biological question and study design, and consulting experienced bioinformaticians and biostatisticians prior to data collection is highly recommended to ensure appropriate data handling and analysis. 

The last step of the workflow is data integration and interpretation, which allows the investigator to link detected compounds with their biological context using publicly available software tools and databases that further enable pathway and enrichment analysis, metabolite/lipid mapping and visualization. Among these available databases, the Kyoto Encyclopedia of Genes and Genomes (KEGG), LIPID MAPS and MetaboAnalyst are widely used in applications of metabolomics and/or lipidomics [48,55]. However, it is important to note that, as highlighted by Schwaiger et al., the specifics of each step within metabolomics and lipidomics workflows can vary significantly [56]. It is also important to emphasize that data integration and interpretation is a step where the investigator’s knowledge of the research field and existing principles, along with deduction skills and deep analysis of the available literature are critical to converting algorithm-generated data into biochemical and physiological insights.

## 3. Metabolomic and Lipidomic Analyses of Acute Exercise-Regulated Biological Networks 

Following the introduction of metabolomic and lipidomic approaches, analytical platforms and experimental workflows above, we overview in this section metabolomic and lipidomic findings made over the last decade in the context of acute exercise. We selected 25 primary research articles and one systematic review specifically focusing on molecular metabolic responses to a single bout of exercise (i.e., acute exercise) within the first minutes/hours and up to 72 h following this single exercise bout, in healthy subjects. Articles that exclusively investigate the effects of acute exercise on the metabolome/lipidome in subjects with disease states (e.g., obese and/or insulin resistant), as well as articles investigating the effects of repeated exercise bouts (i.e., exercise training), were not included in this review. We discuss metabolomic/lipidomic findings related to both acute aerobic and acute resistance exercise bouts, with the term “endurance” used to define an aerobic exercise bout of 30 min or longer duration. As opposed to aerobic exercise which typically consists of repetitive physical activity against relatively low loads and requires the use of oxygen for energy conversion, resistance exercise consists of muscle contractions performed against relatively high loads [57,58]. Findings from both humans and other mammalian species are described, with the aim of highlighting how metabolic networks are affected by exercise in several biological fluids and tissues (mainly skeletal muscle and liver) and setting the stage for future expansion of exercise’s molecular landscape. Collectively, these findings emerge from the use of multiple analytical strategies (i.e., targeted and untargeted) and platforms, with MS-based analytical platforms predominantly being used. See Tables S1 and S2 for further experimental details and summaries of findings from each study discussed below involving metabolomics and lipidomics, respectively.

### 3.1. Metabolomic Analyses of Acute Exercise

#### 3.1.1. Humans

##### Biofluid Analyses

An acute bout of exercise dynamically impacts the human metabolome in a range of biological fluids including blood, plasma/serum, urine and sweat, amongst others. The high variability between existing human studies in terms of age, sex, BMI, exercise type (resistance versus aerobic/endurance), modes (duration, intensity, interval versus continuous), sample types, collection time points, as well as analytical platforms used, presents challenges in distilling these large datasets into a consensus molecular metabolic signature of exercise. 

Blood is considered an integrative biofluid given that it contains metabolites exchanged between organs and is therefore suitable for relatively comprehensive metabolic profiling [10]. Blood collection is relatively non-invasive and available in sufficient amounts for metabolomics/lipidomic purposes in humans. Compared to blood, the urinary metabolome is less comprehensive and complex (i.e., lower metabolite coverage, mostly hydrophilic compounds) since it is a filtrate of wastes from the bloodstream [59]. However, urine is an attractive biological matrix since it can be collected non-invasively in large volumes, and is under no homeostatic control mechanisms, meaning that urine may magnify some metabolite changes occurring in blood. Urine is therefore often used as a matrix for dietary intake biomarker discovery and drug or doping testing [59,60]. Similar to urine, sweat, which is made of ~99% water, mainly contains hydrophilic compounds in addition to electrolytes. Compounds such as proteins, peptides and amino acids, but also urea, lactate and pyruvate can be found in sweat, as well as xenometabolites such as drugs and cosmetics [60]. Researchers should consider which biofluid is practical and ensure developed standard operating procedures exist in order to minimize the wide variety of artifacts which can influence metabolite measurement. The choice of a suitable biological matrix to investigate in the context of exercise will therefore come down to the nature of compounds of interest (hydrophilic and/or hydrophobic compounds), research question (e.g., comprehensive profiling versus specific submetabolome characterization, drug and doping testing), feasibility and experimental setting (e.g., multiple sampling, required volumes, risks of contamination, sample handling, field or sport setting versus laboratory-based). Future advancements in analytical methods may promote development of technologies which can be routinely deployed to capture metabolites from biofluids such as sweat and saliva, for example in an elite sport setting, which will ultimately complement gold standard measures of metabolites from plasma and serum.

Following a qualitative systemic review of human exercise metabolomics studies by Sakaguchi et al. [61], Schranner et al. conducted a recent systematic review of human metabolomic analyses assessing metabolite trajectories following acute endurance and resistance exercise interventions with a duration ranging from 30 min to ~9 h [62]. This systematic review addressed some of these challenges by analyzing a total of 27 studies meeting eligibility criteria, revealing significant changes in up to 196 metabolites in the first 24 h following a single exercise bout. These changes in metabolite concentrations were summarized in the early (0–30 min), intermediate (>30 min–3 h) and late (>3–24 h) stages post-exercise, and divided into classes including: carbohydrates and TCA cycle intermediates; fatty acids (FA), acylcarnitines, ketone bodies, membrane lipids; amino acids and derivatives; and nucleotides, vitamins and co-factors. 

Despite some metabolite classes such as amino acids and derivatives showed mixed responses (i.e., both increased and decreased relative abundance) between exercise types (i.e., resistance versus endurance) as well as differences between endurance studies amongst the 27 studies analyzed [62], other metabolites exhibited robust unidirectional changes following a single exercise bout. Among these, lactate and pyruvate—two well-documented end products of glycolysis—expectedly increased to various extents in the early stages after both acute endurance and resistance exercise. Likewise, several components of the TCA cycle were commonly increased in blood and urine in the early and intermediate post-exercise phases. Among the observed increases in metabolite abundance following exercise, some nucleotides and their degradation products such as hypoxanthine and inosine were also commonly detected. However, the most robust changes in response to exercise involved fat metabolism. Indeed, no less than 37 FA and 17 acylcarnitines were consistently reported to be increased following acute endurance exercise. Acylcarnitines are FA bound to carnitine, an amino acid derivative which allows the transport of FA into the mitochondria where they can be oxidized and contribute to cellular energy conversion. However, acylcarnitines can also accumulate and be released by cells into the bloodstream. Most studies (predominantly endurance exercise studies) reported in this systematic review showed increased levels of several ketone bodies, along with reduced levels of ketogenic amino acids such as leucine, isoleucine and lysine, and increased levels of degradation products from these three amino acids. Conversely, membrane lipids and bile acids were mainly observed to be decreased following acute endurance exercise. Mixed responses were observed for other metabolite classes such as steroid hormones, some vitamins, co-factors and exogenous compounds in addition to amino acids and derivatives following endurance exercise. 

The mixed responses observed in amino acid levels following an acute exercise bout [62] may be explained by the fact that a wide range of exercise types, durations and intensities (Figure 1) and various biofluids with varying sample collection time points are often analyzed together in such systematic reviews to compare exercise with a control resting condition. Amino acid responses can vary in multiple ways, depending on these exercise variables. For instance, if a strenuous endurance exercise bout exceeds the carbohydrate store of an individual, or if the individual’s maximal FA oxidation capacity is reached, a shift towards protein catabolism and amino acid utilization to sustain energy requirements during the prolonged exercise bout will eventually result in reduced circulating amino acid levels [63,64]. Similarly, circulating levels of amino acids can also decrease in the recovery phase following resistance exercise, characterized by an increased utilization of amino acids for protein synthesis. Indeed, all amino acids measured in blood following acute resistance exercise in this systematic review were decreased, except for alanine which was increased. These metabolite changes during the recovery phase may not be observed following less intense and/or shorter exercise bouts. Additionally, a wide range of blood collection timings during and/or following an exercise bout may represent different fasting/feeding periods (often not controlled for in human studies) that will have a major impact on relative circulating amino acid concentrations observed between studies. Furthermore, the fact that amino acids are involved in various metabolic reactions represents another potential reason for mixed amino acid responses to different exercise stimuli. For example, amino acids are involved in protein synthesis, ATP synthesis, gluconeogenesis and ketogenesis. While these reactions will lead to reduced circulating amino acid levels, other reactions such as protein breakdown or dietary protein intake will conversely increase circulating amino acid levels. Intake of carbohydrates will impact amino acid metabolism, as increased carbohydrate availability will inhibit gluconeogenesis and ketogenesis, therefore reducing the utilization of ketogenic and gluconeogenic amino acids to facilitate these metabolic processes [65,66]. From this seminal systematic review [62], depicting differences in metabolomic behaviors between resistance and endurance exercise is limited given the current scarcity of studies investigating the metabolomic responses to a single bout of resistance exercise. Therefore, the only reported metabolite with a clear opposite behavior between acute endurance versus resistance exercise reported is the ketone body acetoacetate, which is increased after endurance exercise while decreased after resistance exercise. The reader should however be aware that differential responses between exercise types, intensities and durations may also lie in the magnitude of metabolite responses rather than directionality. In summary, nutritional status, exercise types and modes represent important confounding factors between studies, introducing challenges and potentially limiting the interpretation of metabolomic responses to acute exercise. This warrants further efforts to characterize amino acid metabolism in response to different exercise types and modes, with particular attention to these common confounders.

- Blood

Overall findings from this systematic review [62] are supported by previous work investigating the human serum metabolome in male and female athletes in response to marathon running. Stander and colleagues reported increased serum FA, ketone bodies and TCA cycle intermediates, along with decreased levels of amino acids following marathon running [64]. Increased concentrations of carbohydrates and associated metabolites, as well as elevated alpha-hydroxy acids and odd-chain fatty acids (OCFA) were also observed following acute endurance exercise [62,64]. The presence of elevated alpha-hydroxy acids and OCFA levels are indicative of an increased utilization of α-oxidation—the process resulting in removal of the carboxyl group (the first carbon atom) in a FA— resulting in the generation of OCFA, though OCFA can also be provided through the diet (e.g., dairy products) [67]. In this study [64], FA α-oxidation was suggested as a potential alternative pathway for energy conversion when β-oxidation reaches saturation, indicated by the accumulation of 3-hydroxy acids (β-hydroxyhexanoic acid) and 3-keto acids (β-hydroxy-α, β-didehydrosebacic acid). Although the peroxisome—the site of α-oxidation—does not contain a TCA cycle nor an electron transport system and is therefore unable to directly produce ATP, the α-oxidation of FA generates alpha-hydroxy acids which can be further subjected to β-oxidation in the peroxisome [68]. These products of the peroxisomal β-oxidation could potentially be taken up by mitochondria for complete oxidation in humans, similar to what has been demonstrated in rodent skeletal muscle [69]. 

In contrast to some of the above findings, other studies have identified elevated serum levels of amino acids including alanine, tyrosine and phenylalanine in males following marathon running, whereas alterations of cholesterol and steroid metabolism following a marathon were consistently reported in these two studies [64,70], as elevated levels of squalene and pregnenolone are indicative of cholesterol breakdown. Pregnenolone is notably a precursor of cortisol, a known steroid stimulator of lipolysis, protein breakdown and gluconeogenesis [71]. In addition, marathon running in these males also provoked decreased serum levels of glucosamine [70]. Glucosamine is a compound involved in joint and cartilage structures, and commonly used as a dietary supplement to combat joint inflammation [72]. Next, caffeine metabolism was also shown to be increased by marathon running, indicated by increased levels of compounds including theophylline, theobromine and xanthine [70]. It is plausible that the increased levels of caffeine and associated derivatives are due to dietary caffeine intake in the hours preceding the marathon, since diets between baseline blood collection (day preceding the race) and the race day were not controlled in this study.

Metabolomic responses to exercise in blood have also been shown to be influenced by an individual’s level of fitness. For instance, in a study by Schader and colleagues [73], male amateur marathon runners were divided into top (*n* = 18), average (*n* = 40) and low (*n* = 18) performers, based on VO_2_max (~63, 50 and 42 mL × min^−1^ × kg^−1^, respectively) and race completion time (~175, 225 and 277 min, respectively). Blood samples from these three groups were examined for potential differences in metabolomic responses to marathon running. Immediately post-race, the low performers exhibited a significant increase in a wide range of acylcarnitines (from short to medium- and long-chain) in plasma compared to the average and top performers groups. One possible explanation for these differences in acylcarnitine levels between top/average and low performers is that low performers may have a reduced capacity to oxidize lipids, which may in turn lead to an accumulation of acylcarnitines. Differences in arginine metabolism and urea cycle related-metabolites between low and top performers have also been reported [73]. Arginine is an amino acid central to the urea cycle, which is activated during protein breakdown when nitrogen is liberated from amino acids. Ornithine, a co-product of urea production from arginine, was shown to be lower in the top versus low marathon performance group, whereas citrulline—an alternative product of arginine metabolism—increased in the top performers group. A potential explanation for the reduced plasma levels of ornithine in top versus low performers may be the greater lactate production in these faster runners; lactate being an inhibitor of urea (and ornithine) synthesis [74]. Furthermore, it is plausible that reduced citrulline levels in low versus top runners can result from increased nitric oxide (NO) production due to longer race duration. NO has been shown to exert a negative feedback regulation of NO synthase, an enzyme that catalyses the production of citrulline and NO from arginine [75]. Another possible reason for reduced levels in citrulline observed in the low performers group may be the potential decrease in asymmetric dimethylarginine—a substrate for citrulline synthesis—that occurs during strenuous and prolonged exercise [76]. These overall differences in the urea cycle and arginine related-metabolites indicate a higher reliance on protein catabolism in low compared to top marathon performers [73]. However, given the complexity and multiple possible reactions leading to the production of arginine and urea cycle related-metabolites, further investigations are required to confirm the potential underlying mechanisms suggested above.

Another recent metabolomics analysis of plasma from young active men performing an acute time-to-exhaustion cycling trial allowed the separation of metabolomic profiles in a 20-min window, and the identification of biomarkers at the onset of fatigue [77]. One key finding from this study was that several metabolites permitted the discrimination between the pre- and post-fatigue states. In this study, Manaf et al. also revealed FA were among the strongest metabolomic responses to exhaustive exercise and progressively increased over time throughout the cycling trial. Particularly, robust increases in oleic and palmitic acids, as well as their carnitine-bound form, were observed while tryptophan concomitantly decreased. This supports the central fatigue hypothesis proposed by Newsholme et al. which suggests that increases in FA levels induce a displacement of tryptophan from albumin, resulting in enhanced availability of free tryptophan. The latter can thus enter the central nervous system to produce serotonin, a neurotransmitter associated with fatigue when it accumulates in the central nervous system [78]. This hypothesis is further supported by the observed increased levels of the end product of serotonin metabolism, 5-methoxy-3-indoleacetic acid. Other potential mechanisms implicating the aforementioned FA and acylcarnitine in the onset of fatigue have also been suggested, including inhibitory effects on adenine nucleotide translocase, responsible for the transport of ATP from the mitochondria to the other cellular compartments requiring energy [77,79].

Recently, Contrepois et al. investigated metabolic responses to acute aerobic exercise (i.e., ~8–12 min of treadmill running following warm-up) using multi-omics (including proteome, transcriptome, metabolome and lipidome) blood profiling [27]. The study investigated multiple biological layers before exercise and at four time points (2, 15, 30 and 60 min) following a single exercise bout. Plasma was collected from healthy older participants (i.e., average age 59 years) with wide ranges of insulin sensitivity and metabolic health status. In line with the recent systematic review discussed above [62], these metabolomic data showed robust lipolysis and FA tissue uptake in response to exercise, indicated by large increases in various FA and acylcarnitines. However, distinct trajectories were observed depending on FA and acylcarnitine carbon chain length and the number of unsaturated bonds. While most saturated medium-chain acylcarnitine (C6:0 to C12:0) levels increased immediately post exercise to return to pre-exercise levels within 15–30 min, several monounsaturated medium- to long-chain acylcarnitines (C6:1, C8:1 and C16:1) and one saturated medium-chain acylcarnitine (C14:0) showed a more modest accumulation with exercise, but returned to pre-exercise levels after 30 to 60 min of recovery. Increased circulating levels of medium-chain acylcarnitines likely suggest incomplete FA oxidation within tissues such as skeletal muscle. Expectedly, free carnitine levels exhibited inverse trajectories, as free carnitine binds to FA to form acylcarnitines. Three main trajectories were observed for FA. While C10 and C12 FA increased two min post-exercise, C14 to C18 FA peaked at 15 min post-exercise, whereas C20 to C24 FA rapidly decreased post-exercise. In this context, the rapid drop of circulating long-chain FA likely indicates increased skeletal muscle uptake of these specific FA during exercise for subsequent oxidation. Distinctively, increased circulating levels of C10 to C18 FA in the first 15 min of recovery may potentially be explained by FA uptake and oxidation switching off more rapidly than exercise-induced lipolysis [80]. As opposed to FA, most amino acids such as glutamic acid, cystine, tryptophan, serine, threonine and glycine decreased within two min of recovery with a return to basal levels by 60 min of recovery. Alternatively, circulating BCAA levels exhibited a delayed decrease following exercise and remained reduced at 60 min of recovery. However, increases in alanine and tyrosine, in line with previous work [70], as well as increases in glutamine and proline were observed, with a return to basal levels within 60 min [27]. The precise reasons for these mixed amino acid responses in blood remain to be elucidated, although increased plasma alanine and glutamine levels indicate ammonia detoxification [27,63].

Interestingly, metabolomics analyses of blood samples have also proven useful in studying the role of liver in exercise metabolism. Indeed, Hu et al. recently highlighted liver-skeletal muscle crosstalk during acute exercise by analyzing arterio-venous differences of metabolites in: (1) the hepato-splanchnic bed; and (2) the exercising and resting leg, in young men [81]. These data indicated only minor changes in saturated long- and very long-chain FA, whereas C6:0 and C8:0 FA, as well as TCA cycle intermediates (succinate and malate), were released by the exercising leg and taken up by the liver through the hepato-splanchnic bed. Blood analyses from the hepato-splanchnic bed therefore represent a means to study liver metabolism in humans while avoiding challenges associated with the invasive nature of liver biopsy collection.

- Urine

Although blood is the most commonly studied biofluid in the research area of exercise metabolomics, an increasing body of work in the field of exercise involves analysis of other biofluids including urine, saliva and sweat. After blood, urine is seemingly the most commonly analyzed biofluid in the context of exercise metabolomics. In humans, metabolite profiling of urine samples is appealing since it has proven to be more stable, under less homeostatic regulation than other biofluids [59], and collected non-invasively and in larger volumes compared to other biofluids. It has been suggested that the urinary metabolome can be considered complementary to the blood metabolome, since urine contains numerous end-products derived from food and drug metabolism [50]. Recent publications support the utility of urine analysis to reflect metabolomic changes following acute exercise, as analysis of urine permits confirmation of well-appreciated exercise-induced changes in metabolites related to several pathways including glycolysis (e.g., pyruvate and lactate), TCA cycle (e.g., citrate and succinate) and amino acid metabolism (e.g., alanine, taurine) [82,83,84,85,86]. In one of the earliest urinary metabolomic papers published, Kistner et al. reported that within 15–30 min following an incremental cycling test, significant increases in urinary carnitine and novel urinary exercise-responsive metabolites could be observed; notably including increases in leucine derivatives methylsuccinate and 3-hydroxyisovalerate, and valine derivative 3-aminoisobutyrate. Increased urinary levels of these derivatives indicate branched-chain amino acid (BCAA) degradation and excretion in urine following exhaustive exercise [82]. However, an often-reported downside of metabolomic analyses in urine is that metabolite concentrations are highly influenced by hydration status and thus require normalization for water content. Several pre-acquisition normalization methods have been developed to address these issues. The most popular methods, each presenting advantages and drawbacks, include the assessment of relative concentration to a reference compound such as creatinine, measurement of osmolality, and the assessment of urine specific gravity (i.e., urine to pure water density ratio) [50].

- Saliva

Saliva has gained attention over the past few years in the study of exercise metabolomic biomarkers. Like urine collection, saliva is collected non-invasively and does not require specialized laboratory facilities or skilled healthcare professionals. However, only few metabolomics-based studies investigating the effects of exercise in saliva have been conducted to date (e.g., [87,88,89,90,91]), and several potential pitfalls have been underscored. The salivary metabolome contains both metabolites from the body and oral bacteria, as well as ingestion-related compounds. It has also been observed that some metabolites such as lactate return to basal states much faster in saliva versus blood [92]. Similar to urine, metabolite concentrations are substantially affected by hydration status, therefore also requiring normalization for water content. Normalization based on total protein concentration of whole saliva (TPWS) and total observed metabolite concentration (TOMC) have been suggested to address this issue. However, normalization for water content in metabolomics studies in saliva has not been systematically performed to date [89]. Additional efforts are therefore needed to use saliva as a reliable source of biological information in the exercise research field.

- Sweat

Sweat also represents an understudied biofluid in the field of exercise metabolomics, partly due to its relatively low metabolite concentrations. However, sweat metabolomics has proven useful in other contexts such as cancer diagnostics [93]. Among metabolomics-based exercise studies performed in sweat (e.g., [94,95,96]), in a pilot study Harshman and colleagues [95] identified dozens of metabolites following a treadmill march with 22-kg tactical gear until perceived exhaustion, either at low (4.8 km/h, 3% incline), moderate (5.1 km/h, 4% incline) or high intensity (5.6 km/h, 6% incline), in active duty military volunteers. Consistent with previous findings, amino acids were the predominant detected compounds [94,96]. However, the authors failed to observe any significant changes in metabolite concentrations between conditions, and no correlations could be drawn between metabolite concentrations and aerobic capacity (VO_2_max) or the rate of perceived fatigue. Several confounding factors and current pitfalls of sweat-based metabolomics studies have been reported, including the absence of localized sweat rate measure; normalization methods of analyte concentrations; sweat collection devices utilized, which can also constitute a great source of interindividual and inter-study variability; and the presence of skin bacteria and cosmetics that may interact with sweat metabolites [95]. This, in combination with the frequent lack of statistical power in these human studies, limits the full potential of sweat metabolomics and questions whether sweat is a reliable biofluid for exercise biomarker discovery purposes. 

#### 3.1.2. Other Mammals and Tissues

Overall, metabolomics studies analyzing human biofluids in the context of exercise are far more common than human studies investigating tissues. One of the main reasons for the currently limited tissue metabolomics studies involves the more invasive nature of human tissue biopsies (e.g., skeletal muscle and liver) compared to routine blood sampling or sweat, urine and saliva collection. However, it has been demonstrated that blood and skeletal muscle metabolomes have very little overlap, thus suggesting an overall limited ability to potentially identify muscle tissue-specific metabolites from blood samples [97]. Mammalian animal models have therefore helped expand our understanding of metabolic networks affected by acute exercise by allowing easier access to metabolically active tissues such as skeletal muscle and liver. 

As opposed to human studies, other mammalian studies (e.g., mice, rats) assessing the effects of a single bout of exercise on the metabolome have predominantly analyzed tissues relative to biofluids. These biofluids including saliva, sweat and urine are not collected as easily and not available in sufficient volumes in small mammals such as rodents. Several metabolomics studies in rodents have analyzed skeletal muscle following an acute bout of exercise. Building upon the results in human biofluids demonstrating differential responses to exercise depending on fitness level and performance, it has been shown in mouse hindlimb skeletal muscle that metabolic responses to exercise are dependent on the time of the day during which exercise is performed [98]. In a study from Sato et al., mice were subjected to a treadmill running bout either in the early active phase or the early rest phase (equivalent to early morning and late evening in humans, respectively), and their hindlimb skeletal muscles were subjected to metabolomics analysis. These results suggested an increased glucose utilization, along with increased use of other fuel sources such as lipids, amino acids and ketone bodies, when mice exercised in the early active compared to the early rest phase [98]. 

Furthermore, metabolomic analysis of both plasma and hindlimb skeletal muscle from rats with high and low running capacities has provided insights into substrate utilization during and following an exhaustive running bout [99]. Following a 10-min run (i.e., exhaustion for low-performance rats), only marginal increases in skeletal muscle long-chain acylcarnitines were observed in low-performance running rats, with very little changes observed in plasma FA. Conversely, high-performance rats exhibited significantly increased muscle levels of these long-chain acylcarnitines together with reduced plasma FA, indicating enhanced FA muscle uptake. Only following exhaustion in high-performance rats (45 min) were medium- and long-chain acylcarnitines increased both in muscle and blood. While increased long-chain acylcarnitines in muscle and blood potentially indicate that FA oxidation capacities were reached, increased circulating levels of medium-chain acylcarnitines likely indicate incomplete FA oxidation [80]. Decreased plasma and muscle levels of BCAAs in high- versus low-performance rats were also observed at 10 min, suggesting increased BCAA uptake and breakdown within the skeletal muscle. The data from this study overall showed enhanced FA and BCAA utilization capacities in high- versus low-performance rats. While the increased FA oxidation capacities in high-performance rats are in line with findings from human marathon runners [73] described above, the amino acid results between these studies seem contradictory. However, the absence of significant changes in both plasma and muscle amino acids in low-performance rats may be due to the relatively short exercise bout (10 min) that may have been insufficient to induce protein breakdown as opposed to the 45-min run in high-performance rats. Of note, the mechanisms of exhaustion were similar between low and high capacity running rats, with exhaustion only being delayed in high capacity running rats.

In addition to rodents, tissue metabolomic responses to acute exercise have also been studied in other mammalian species. For instance, the skeletal muscle metabolome in horses was recently investigated after a single incremental exercise test to exhaustion, in both an untrained and trained status [100]. Only 31 of all identified metabolites were changed 3 h following the treadmill race in untrained horses, while 142 metabolites significantly changed in the trained horses. Regardless of training status, the predominant exercise-induced response to acute exercise involved changes in amino acid (including BCAAs) and lipid metabolism. Nucleotides and xenometabolites also showed altered levels in horse skeletal muscle following the exercise bout. Given the increased number of metabolites significantly altered in the trained state, the authors suggested that interindividual variability can be attenuated by training. This may also reflect the enhanced ability of trained horses to run at higher intensities for a longer amount of time. However, it is important to note that post-exercise muscle biopsies were collected 3 and 24 h following the exercise test. Although food was only allowed after the 3-h post-exercise biopsy, the sample collection delay represents a limitation to this study that may have hindered observation of additional exercise-induced metabolite changes in both trained and untrained horses. Moreover, a noteworthy point raised by Zhang and colleagues [101] is that muscle biopsies do not allow distinction between extra- and intra-muscular metabolites. To do so, investigating skeletal muscle interstitial fluids is a promising, yet rarely practiced avenue in metabolomics research that has the potential to provide more accurate insights into mammalian muscle metabolism. For example, a recent study assessed both plasma and muscle interstitial fluids in rats following a short treadmill running bout at moderate intensity. Out of 299 detected metabolites, only 43% were common to both biofluids. Among the 204 metabolites changed by exercise, only 20% were shared, therefore underscoring the limited ability of circulating metabolites to reflect the full range of muscle metabolic changes induced by exercise [101]. Additional pilot data from human muscle interstitial fluid was also collected in this study and, in line with rat data, reported increased TCA cycle intermediates following exercise, possibly induced by increased FA oxidation. Likewise, increased levels of amino acids and markers of purine catabolism were observed, among others, following the exercise bout. Of note, differential FA responses were observed between rats and human data, potentially resulting from differences in sample collection timing. Taken together, these results warrant further mammalian metabolomics-based investigations in multiple tissues and/or biofluids to potentially capture a more detailed molecular blueprint of acute exercise metabolism.

The liver, which is crucial to whole-body energy supply and maintenance of metabolic homeostasis during exercise, has also been studied using metabolomic approaches. In 2010, Huang and colleagues were the first to use metabolomics to investigate changes in liver metabolic profile induced by exhaustive treadmill running in rats [102]. Increased hepatic levels of xanthine, hypoxanthine, and creatinine—a degradation product of creatine—point to increased energy conversion, while decreased hepatic levels of carbohydrates and lactate suggest glycogen depletion, to help meet exercise-induced energy requirements. Increased FA and ketone bodies further support an increased reliance on fat metabolism during prolonged exhaustive exercise. Additionally, this was accompanied by increased urea concentrations. Exhaustive exercise was associated with hepatic inflammation, as elevated levels of inflammatory precursors such as arachidonic, linoleic and oleic acids were detected and associated with the accumulation of macrophages detected by liver immunochemistry [102]. As previously discussed, Hu and colleagues who observed that FA (C6:0, C8:0) and TCA cycle intermediates (succinate and malate) were released by the exercising leg and taken up by the liver [81], conducted complementary analyses on liver transcriptome data obtained from mice following a 60-min treadmill running bout [103]. Findings indicated exercise-induced activation of HIF-, NRF2- and cAMP-dependent gene transcription, potentially indicating that metabolites released from the exercising muscle can also act as signaling molecules in the liver [81,103], although it cannot be excluded that activation of liver gene transcription may be driven by the liver’s own amplified metabolism and signaling molecules. It was speculated that these circulating metabolites may be involved in metabolic adaptations to exercise, though it was acknowledged that further research is needed to validate this hypothesis [81]. Although inter-organ crosstalk during exercise is still overall poorly understood and requires further investigations, it is important to note that feasibility of liver metabolomic investigations in humans is very limited, given the invasiveness of liver biopsy sampling. Assessing hepato-splanchnic fluxes by collecting blood from hepatic veins and peripheral arteries may help partially address this issue in future human studies [81,104].

### 3.2. Lipidomic Analyses of Acute Exercise

#### 3.2.1. Humans

##### Blood Analyses

The release of vasoactive metabolites from the working skeletal muscle and vascular endothelium during exercise is well documented [105]. These numerous vasoactive substances comprise lipid species, including epoxides (also named epoxy FA) derived from arachidonic acid (AA) such as 5,6-, 8,9-, 11,12-, 14,15- epoxyeicosatrienoic acid (EET) isomers, are produced by the action of cytochrome P450 (CYP) mono-oxygenase (Figure S1) [106]. EETs can in turn induce hyperpolarization of smooth muscle cells, leading to vascular relaxation, which has been suggested to contribute to enhanced skeletal muscle blood flow during exercise [107]. Recently, it was demonstrated that vasoactive lipids derived from the *n*-3 and *n*-6 polyunsaturated FA (PUFA) and metabolized by CYP were also released into the bloodstream following an acute maximal treadmill test using the Bruce protocol [108] in healthy adults [106]; and 12,13- epoxyoctadecenoic acids (12,13-EpOME)—an epoxide originating from linoleic acid (LA)—exhibited significantly increased levels in plasma following the maximal exercise bout. Epoxides can be further metabolized to diols by soluble epoxide hydrolase (sEH) (Figure S1). As such, increased plasma levels of diols were reported post-exercise; 5,6-dihydroxyeicosatrienoic acids (5,6-DHET), derived from AA; as well as 5,6- and 17,18- dihydroxyeicosatetraenoic acids (5,6- and 17,18-DiHETE), derived from EPA [106]. Diols such as DHETs, although initially thought to be inactivation products of EETs, also exhibit vasodilation properties [109], potentially counteracting vasoconstrictive substances concurrently released during exercise. In contrast to EETs and DHETs, EpOMEs (especially 12,13-EpOME) have been shown to have cardiac depressant and vasoconstrictive properties [110]. The physiological roles of 5,6- and 17,18-DiHETE are largely uncharacterized, although its upstream epoxide 17,18-epoxyeicosatetraenoic acid (17,18-EEQ) is another vasodilator [111]. 

While no significant changes in plasma levels of other detected epoxides and diols were observed following maximal exercise in humans, Stanford and colleagues recently showed substantially increased levels of circulating 12,13-dihydroxyoctadecanoic acid (12,13-DiHOME)—the downstream product of 12,13-EpOME—after a moderate-intensity exercise bout (cycling and running at 70 and 75% VO_2_max, respectively) in healthy humans, regardless of sex, age and physical activity level [112]. In this same study, the authors also showed that exercise increases circulating 12,13-DiHOME in male mice and that this increase was negated by the surgical removal of brown adipose tissue (BAT), indicating that in mice, 12,13-DiHOME is released from BAT during exercise. Furthermore, mice injected with 12,13-DiHOME had higher skeletal muscle FA uptake compared to mice injected with vehicle control, and mouse myotubes incubated with 12,13-DiHOME displayed increased FA uptake and oxidation [112]. These lipidomic findings highlight a crosstalk between adipose tissue and skeletal muscle during acute exercise and identify potential factors that may contribute to some metabolic health benefits of exercise [112]. However, a wide range of functions have been attributed to 12,13-DiHOME including detrimental effects on both cardiac [113] and mitochondrial function [114]. The biological meaning of increased DiHOMEs thus remains unclear, warranting further investigation. 

Increased plasma levels of 12,13-DiHOME and other lipid mediators have also been observed in plasma collected from trained male cyclists. In this study, cyclists completed a 75-km cycling bout at moderate intensity (~70% of VO_2_max) [115]. Increases in plasma concentrations of 9,10-DiHOME as well as 9- and 13-hydroxy-octadecadienoic acid (9- and 13-HODE) were observed. HODEs are peroxidation products of the *n*-6 LA (Figure S1) that have been linked to oxidative stress, inflammation, physiological and pathological states including atherosclerosis [116]. Although not associated with increased inflammation markers in this study, 9- and 13-HODE were significantly correlated with F_2_-isoprostanes, indicators of oxidative stress (although in much lower abundance), supporting the inclusion of 9- and 13-HODE as oxidative stress biomarkers [115].

Gollash and colleagues have helped expand the understanding of metabolic responses to acute maximal aerobic exercise by investigating changes in lipid profiles from red blood cells (RBC), a constituent of blood largely overlooked in metabolomic/lipidomic studies [117]. RBC represents a reservoir of lipid species including epoxides which can regulate vascular capacity, as previously mentioned. Following maximal acute exercise to exhaustion (see [106]) in healthy non trained adults, venous RBC exhibited increased levels of epoxides including 9,10- and 12,13-EpOME; 5,6-EET, 11,12-EET and 14,15-EET; but also epoxides derived from docosahexaenoic acid (DHA), 16,17- and 19,20- epoxydocosapentaenoic acids (16,17- and 19,20-EDP). These two DHA-derived epoxides have shown vasodilating and cardioprotective properties [118]. All the aforementioned epoxide mediators are generated by CYP mono-oxygenase. In contrast, no changes in lipid mediators generated by lipoxygenase (LOX) and cyclooxygenase (COX) were observed (Figure S1). This suggests that CYP mono-oxygenase-derived epoxides accumulate in RBCs and may, when released, contribute to cardiovascular responses to acute exhaustive exercise [117]. However, no changes in RBC levels of the 20 quantified FA were found following the same exercise protocol [119]. The omega-3 quotient—the percentage of EPA + DHA in FA from RBC membranes—was also unchanged. A low omega-3 quotient (or index) represents an independent risk factor for cardiovascular diseases and increased mortality [120,121]. While short duration maximal exercise was not able to elicit immediate changes in plasma and RBC FA levels (including omega-3 quotient), RBC levels of lauric acid (C12:0) significantly decreased between exhaustion and recovery, 10 min later. Lauric acid may therefore regulate cardiovascular and metabolic functions, and further research is warranted to address this possibility [119].

Efforts to characterize lipid mediators derived from COX, LOX and CYP pathways responding to acute resistance exercise have also been performed [122]. Using targeted lipidomics, 87 lipid species were detected in the serum of 16 young men who undertook a single session of high intensity resistance exercise. The resistance exercise protocol consisted of a circuit of three sets of 8–10 repetitions of leg press, bar squats and knee extension performed at 80% of individual one-repetition maximum (1-RM). Serum was collected before exercise and every 30 min within three hours post-exercise, and then at 24 h of recovery. A wide array of lipid mediators derived from COX, LOX, and CYP pathways were dynamically changed following exercise, including AA-derived metabolites such as prostaglandins (PGs), thromboxanes (TXs) and leukotrienes (LTs). PGs and TXs are formed under the action of COX enzymes while LTs are produced via LOX enzymes (Figure S1). TXs comprise TXA_2_ and its metabolites TXB_2_ and 12-hydroxyheptadecatrienoic acid (12S HHTrE). TXB_2_ and 12S HHTrE are considered biomarkers of TXA_2_ biosynthesis and are more easily detected since TXA_2_ is rapidly degraded into TXB_2_ or 12S HHTrE [123]. PGs comprise four primary compounds: PGD_2_, PGE_2_, PGF_2α_ and PGI_2_. Likewise, PGs are also rapidly converted to primary 6 or 15-keto and secondary 13,14-dihydro-15-keto metabolites. Next, LTs are composed of LTA_4_ and derivatives including LTB_4_ and anti-inflammatory/pro-resolving lipoxins LXA_4_ and LXB_4_. 

Early resistance exercise responses (0–3 h of recovery following exercise) comprised increased levels of (1) TXB_2_ and 12S HHTrE; (2) PGD_2_, PGE_2_ and its derivative 15-keto PGE_2_, 15-keto PGF_2α_, 6-keto PGF_1α_; (3) LTB4 and derivatives LXA4, LXB4; (4) pro-inflammatory AA-derived 12-hydroxyeicosatetraenoic acid (12-HETE) and its byproduct tetranor 12-HETE, along with the anti-inflammatory 15-HETE. Early responses to resistance exercise were also marked with immediate increases in EPA-derived resolvins (RvE1) and DHA-derived 10(S),17(S)-DiHDHA (protectin D1), which are generated by LOX enzymes and exhibit anti-inflammatory properties. In contrast, LOX-mediated LA derivatives such as 9- and 13-HODE and their degradation products 9- and 13-oxo-octadecadienoic acids (9- and 13-oxo-ODE) tended to decrease in the first half hour following exercise before significantly increasing (compared to 30 min post-exercise) and peaking at 2–3 h of recovery. Similarly, CYP-mediated LA metabolite 9,10-EpOME and downstream product 9,10-DiHOME were also elevated at 2–3 h of recovery. Finally, elevations in 11,12- and 14,15-DHET-CYP-mediated metabolites of AA-were also found in the early recovery phase. Most lipid metabolites returned to basal levels within 24 h post exercise, except for a few metabolites which peaked (13,14-dihydro-15-keto PGE2, 6-keto PGF_1α_, 15-HETE and byproduct 15-oxo-ETE, and protectin D1) or remained significantly elevated (12(S) HHTrE). These findings pinpoint the activation of pro-inflammatory and pro-resolving pathways following acute resistance exercise, both in the early and later stages of exercise [122].

In addition to FA-derived lipid mediators involved in pro- and anti-inflammatory/pro-resolving pathways induced by acute exercise, lipidomics has also helped recently uncover other lipid species belonging to other categories and classes and playing potential roles in exercise metabolism. Indeed, the lipidomics arm of Contrepois and colleagues’ work [27] revealed large increases in circulating complex lipids within the early recovery phase (2 min post treadmill run), with a rapid return to pre-exercise levels (15–30 min post). These complex lipids included 23 phosphatidylcholines, 20 cholesteryl esters, 15 triacylglycerols (TAGs), ten diacylglycerols (DAGs), nine ceramides and eight sphingomyelins. In addition, exercise-induced changes in TAGs appeared to depend on carbon number and unsaturation level. For instance, while plasma concentrations of most TAGs (those with shorter saturated FA in particular) decreased at 30 and 60 min of recovery, those which rapidly increased post-exercise (within 2 min) mostly comprised long-chain PUFA including AA, EPA and DHA. Together with the observed early increases in ceramides and sphingomyelins, increased long-chain PUFA suggest activation of both pro- and anti-inflammatory pathways, as demonstrated in previous work [122,124]. In contrast, TAGs with shorter saturated FA may be used as a preferential substrate for energy conversion [27].

##### Tissue Analyses

Similar to the blood analyses previously described, further lipidomics analyses of skeletal muscle have uncovered inflammatory responses to acute resistance exercise. Lipidomic analyses of muscle biopsies from young active men during a single resistance exercise session indicated augmented inflammatory response in the early recovery phase [124]. Two hours post-exercise, substantial increases in skeletal muscle concentrations of various lipid mediators were observed, including COX-mediated TXs and PGs, derived from AA (Figure S1). The recovery period was also marked by increased intramuscular levels of species from the LOX pathways, including derivatives from EPA: 12-hydroxyEPA (12-HEPE); DHA: 7- and 14-hydroxyDHA (7- and 14-HDHA); and AA: 5- and 12- HETE, and LTs. Finally, lipid mediators from the CYP pathway and derived from both AA (5,6-EET; 11,12- and 14,15-DHET) and LA (9,10- and 12,13-DiHOME) were also increased at this timepoint. As mentioned earlier, while a wide range of AA-derived mediators produced through COX and LOX pathways (e.g., TXs, PGs, LTs, 12-HETE) stimulate acute inflammation, several lipid mediators metabolized through the LOX and CYP pathways, (e.g., AA-derived 5-HETE, EETs and DHETs, DHA-derived HDHAs) are potential precursors of pro-resolving mediators that inhibit inflammatory signaling. From this study and consistent with previous work from the same group conducted in serum [122], both pro-inflammatory lipid mediators and lipid markers of pro-resolving mediator biosynthesis are stimulated by resistance exercise simultaneously in skeletal muscle, 2 h post-exercise [124]. 

Lipidomic responses to acute resistance exercise are however influenced by an individual’s age. Applying targeted lipidomics to skeletal muscle from both young (~22 years old) and older (~74 years old) healthy men who undertook a single resistance exercise bout, Rivas et al. observed significant differences in skeletal muscle ceramides between the two cohorts [125]. Ceramides are a subclass of sphingolipids that have emerged as potential modulators of diseases associated with lipotoxicity including IR, type 2 diabetes and cardiovascular disease [126]. Ceramides are also well-recognized activators of proinflammatory signaling [127]. In this study, a relationship was found between intramuscular ceramide levels and impaired exercise-induced anabolic signaling occurring in older men. Older men had significantly higher palmitic (C16:0) and arachidic (C20:0) ceramides levels within skeletal muscle, and a negative correlation between intramuscular C16:0 ceramide and leg lean mass was also observed. Next, blunted anabolic signaling following the exercise bout in older men was associated with increased activation of pro-inflammatory signaling compared to young men. Intramuscular levels of specific ceramides may therefore negatively impact anabolic signaling following a single bout of resistance exercise by promoting inflammation [125]. 

#### 3.2.2. Other Mammals

##### Blood Analyses

In other mammals, to date very few studies have been performed to investigate the effects of acute exercise on the blood lipidome. To our knowledge, the only study aimed to specifically characterize the lipidome following acute exercise in mammals to date is a pilot study performed in Thoroughbred horses [128]. Four horses (3 males and 1 female) were subjected to a supramaximal (115% VO_2_max) treadmill running bout to exhaustion. Of the 933 plasma lipid species detected, 130 were known lipids. Despite the lack of statistical power in this pilot study, 13 lipid species were changed following exercise, including seven FA and six phospholipids; three phosphatidylcholines (PCs), one lysophosphatidylcholine (LPC) and two sphingolipids (SMs). Although not reaching statistical significance, five TAGs decreased by more than 20% following exercise. While six identified phospholipids (i.e., PC (P-34:1), (P-36:2), (P-36:4); LPC (18:0); SM (d36:1) and (d42:2)), along with several FA such as LA, LNA (C18:3), and the *n*-6 11,14-eicosadienoic acid (C20:2), were increased by supramaximal exercise, the remaining FA (C12:0, C14:0, C17:0 and C20:0) were decreased post-exercise. The increase in plasma unsaturated FA may reflect lipolysis in the adipose tissue, reported to contain a higher ratio of unsaturated/saturated TAGs compared to plasma FA [129]. Phospholipids, which also increased immediately post-exercise, progressively decreased over time. As PCs and SMs are major components of cell membranes, the current findings may be explained by the increased cell membrane turnover due to exercise-induced lipolysis and membrane damage. Phospholipids can then be mobilized as energy substrates or for repair of cell membranes damaged during exercise [130,131]. Although this pilot study has important limitations, supramaximal exercise in Thoroughbred horses is shown to affect distinct lipid categories including FA and more complex lipids such as TAGs, SMs and PCs. In addition, these pilot findings will be useful for informing power analyses and sample size requirements in future lipidomic studies [128].

##### Tissue Analyses

As stated previously, the feasibility of investigating liver biopsy responses to exercise in humans is very low; therefore, utilizing other mammalian species has helped gain a deeper understanding of tissue lipidomic responses to acute exercise. In 2010, Hu and colleagues were one of the first to conduct a lipidomic study to uncover molecular responses to acute treadmill running exercise in mouse liver [132]. Among 115 quantified lipid species, PCs and TAGs were the most abundant lipid classes. Almost all detected TAGs tended to increase in the liver immediately post-exercise compared to control rested mice, though statistical significance was not reached. In contrast, a clear difference in hepatic lipid profile was observed following three hours of recovery. The 21 lipid species mostly responsible for lipid profile changes were predominantly TAGs (17), with the remaining lipids being composed of three PCs and one LPC. While all four choline phospholipids decreased (PCs and LPC), all hepatic TAGs increased three hours post-exercise. The significantly increased lipid species included polyunsaturated TAGs (50:3; 54:5; 54:6; 54:7; 56:4; 58:6; and 58:10) and decreased lipids included PCs (36:1; 38:3; 40:4) and DAG (34:1). Although increased TAG concentrations in liver may have been due to refeeding post-exercise (i.e., mice had free access to food during the first two hours of recovery), the authors tested this hypothesis via a fasting/refeeding experiment and observed similar TAG levels in refed mice compared to the rested control mice, while fasted mice had significantly increased hepatic TAGs. The authors therefore concluded that increased hepatic TAGs following three hours of recovery were unlikely caused by food intake. It was rather proposed that the accumulation of TAGs in the liver may result from the large elevation in plasma FA following exercise-induced lipolysis, which may exceed oxidative capacities of the working muscle. The FA in excess may instead be delivered to the liver for transient storage. As opposed to liver, skeletal muscle total TAG content significantly declined immediately post-exercise, suggesting enhanced lipolysis for subsequent FA oxidation [132].

More recently, this same group used a similar endurance exercise protocol to study the lipidomic responses to acute exercise in both liver and hindlimb skeletal muscles (gastrocnemius and soleus) in mice [133]. Their targeted lipidomics data revealed that, while most lipid species detected in mouse liver were unchanged by the single exercise bout, several phospholipids including five LPCs (16:0; 18:2; 20:4; 22:5 and 22:6), four lysophosphatidylethanolamines (LPEs) (16:0; 18:0; 18:1 and 20:4) and two plasmalogen phosphoethanolamines (PE-P) (P-38:4 and P-40:4) increased. The reasons for most of these changes remain to be elucidated. However, since plasmalogens are generally secreted by the liver once synthesized [134], increased hepatic levels of plasmalogens may indicate inhibited secretion with exercise. Free carnitine, acetylcarnitine and 39 acylcarnitines with carbon chain length ranging from 3–20 were also detected in liver, gastrocnemius and soleus muscles. Liver exhibited heightened concentrations of free and total carnitine following treadmill exercise, while acetylcarnitine content was reduced. The detected acylcarnitines were unchanged by the acute exercise bout. Conversely, acetylcarnitine, short-chain acylcarnitines and hydroxy-acylcarnitines were increased following exercise in both skeletal muscles, with acetylcarnitine and hydroxy-acylcarnitines showing much greater increases in soleus (61%) compared to gastrocnemius (15%) muscle. While hydroxy-carnitines are intermediates of incomplete FA oxidation, acetylcarnitine is the end product of the catabolism of all fuels including FA, pyruvate and several amino acids [80]. Higher levels of both acetylcarnitine and hydroxy-acylcarnitines following acute exercise can therefore be explained by the higher FA oxidation rate [135] and the greater carnitine uptake capacities in soleus compared to gastrocnemius muscle [136]. Next, the unchanged levels of medium- and long-chain acylcarnitines after the treadmill run may indicate that this exercise protocol was not sufficiently intense to affect muscle content of these acylcarnitines. In contrast to liver, free and total carnitine in both muscles were not affected by exercise. Increased hepatic concentrations of free and total carnitine upon acute exercise were likely due to enhanced uptake by the liver since the gene expression of the carnitine transporter Slc22a5/OCTN2 was upregulated [133]. Finally, isotope-tracing of FA and acylcarnitines showed that, consistent with previous findings [132], excess circulating FA are taken up by the liver and incorporated into TAGs and phospholipids during recovery, highlighting tissue-specific differences in FA uptake [133].

**Figure 3:**
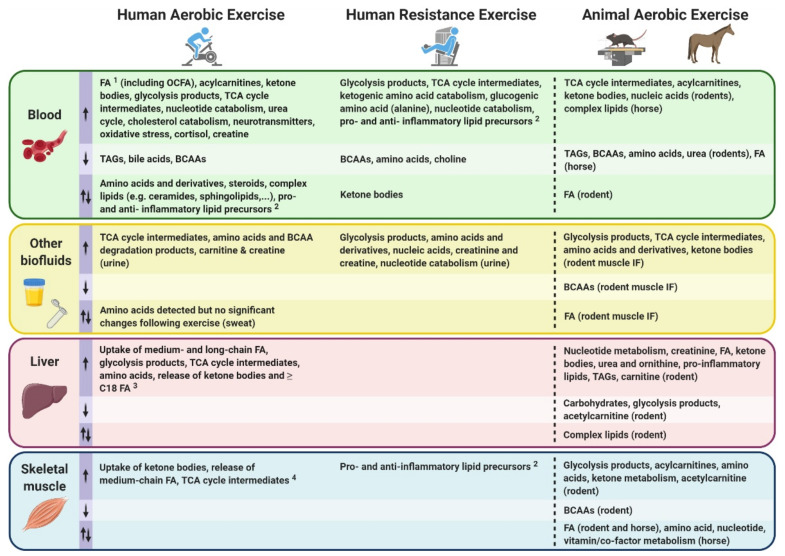
Summary of the current exercise molecular landscape of metabolomic and lipidomic findings discussed from the past decade. Dynamic changes in metabolites and lipids (i.e., increased, decreased, or shown to be changed in both directions) occurring in response to acute aerobic and resistance exercise are summarized across various biological systems (i.e., blood and other biofluids including urine and sweat; and tissues such as liver and skeletal muscle) in humans and other mammalian species. This figure focuses on early responses (0–30 min) following an acute exercise bout except for data collected from horses within the first three hours post-exercise. Metabolomic responses can therefore show different directionality based on timing and exercise variables, as mentioned previously. BCAAs: branched-chain amino acids, FA: fatty acids, IF: interstitial fluid, OCFA: odd-chain fatty acids, TAGs: triacylglycerols, TCA: tricarboxylic acid. ^1^ Changes in FA levels post-exercise may depend on carbon chain length and although circulating levels of most FA have been reported to increase following an acute exercise bout, post-exercise decreases in some FA chain lengths (e.g., C20 to C24) have also been observed. ^2^ Directionality of specific lipid mediators in human blood and skeletal muscle are not detailed in this figure, and responses to exercise may vary depending on the specific lipid mediators within their broader classes. Refer to Table S2 for information regarding the directionality of specific pro- and anti-inflammatory lipid precursors following exercise. The metabolite directionalities of human liver ^3^ and human skeletal muscle ^4^ depicted in this figure are derived from data analyzing hepato-splanchnic bed and arterio-venous differences, respectively, rather than from tissue biopsies. Up arrow: increase; down arrow: decrease; bidirectional arrow: both increase and decrease.

The extensive exercise metabolomics and lipidomics literature from the past decade discussed above has helped build a strong foundation to continue expanding the molecular landscape of exercise in the next decade. Figure 3 summarizes the current exercise molecular landscape by overviewing the dynamic changes in metabolites and lipids that occur in response to acute aerobic and resistance exercise across species (i.e., humans and other mammals) and biological systems (i.e., blood, other biofluids, liver and skeletal muscle).

## 4. Current Challenges and Remaining Knowledge Gaps to Continue Expanding Exercise’s Molecular Landscape 

### 4.1. Metabolite Identification and Annotation

To continue expanding the exercise molecular landscape in the next decade, metabolite and lipid identification/annotation still represents a main challenge and bottleneck of untargeted metabolomics and lipidomics approaches, in contrast to protein identification in proteomics, for example. Whereas proteins are composed of a finite and more manageable combination of different amino acids that can be sequenced by matching experimental peptides against in silico fragmentation spectra, metabolites (including lipid species) are a highly heterogenous group of small molecules resulting from countless different chemical structures and atomic combinations, although predominantly composed of the elements C, H, N, O, P and S [137]. Despite recent technological advances in analytical instrumentation that have enabled rapid and simultaneous detection of thousands of metabolites from very low volumes of biological samples, a much smaller portion of these metabolites can remain after stringent data processing and cleaning processes prior to any attempt at identification/annotation [138]. These data processing and cleaning steps are essential to generate more high-confidence metabolomic and lipidomic datasets, but the overall trade-off is reduced metabolite coverage.

Next, metabolites and lipid features (such as mass-to-charge ratios and retention times) that meet quality control criteria can still correspond to numerous molecular structures. Their identification—a term used when the highest level of confidence is reached; level 1—or annotation (lower level of confidence in metabolite characterization, levels 2 to 3) [51] notably depends on an existing reference match in currently available databases, and preferably an in-house generated database. This is important, as the vast majority of features currently fail to match any metabolite from these databases and are therefore assigned as “unknowns”. These unknowns may be true unknowns (i.e., compounds for which no chemical structure, name, origin, and biological function has been described to date), but some compounds may however be assigned as unknowns because the reference is missing from the available databases. Most existing databases are still largely incomplete, and in the case of true unknown metabolites and/or lipids, extensive efforts in analytical chemistry are required to characterize their molecular structure. However, these characterization efforts are rarely undertaken given their challenging and time-consuming nature [139]. As a result, unknowns within datasets are often disregarded, and attention is instead focused on only putatively named metabolites. In the case of compounds that are matched against a database, additional information is necessary to accurately identify and validate a single candidate since basic features such as retention time and m/z may have multiple candidates. MS^2^ (and sometimes MS^n^) is required to reach the highest level of confidence, as fragmentation patterns help elucidate molecular structures and distinguish metabolites with similar *m*/*z* and retention times by matching them with fragmentation patterns of authentic chemical standards within metabolite libraries. Nevertheless, most libraries are still largely incomplete, therefore the number of authentic chemical standards available represents a current limiting factor to metabolite identification of the broader metabolome. Additionally, compounds can exhibit different levels of confidence in identification/annotation, making data integration and interpretation even more challenging since most commonly used dedicated tools (e.g., KEGG, MetaboAnalyst 3.0) require metabolite identification (i.e., level 1) to integrate the data into biological context [137]. 

Efforts to expand libraries with authentic standards in the next decade will help exploit the full potential of untargeted metabolomics by yielding a much higher coverage of unequivocally identified metabolites. MS^2^ is however more time- and resource-consuming. In addition, validation of metabolite identification/annotation still requires extensive human intervention, since this step is usually performed manually and requires expertise in chemical structure and biochemistry. This hurdle may become a growing issue as the number of metabolites to manually validate increases with the expansion of metabolite libraries in the years to come. It is also important to note that MS^2^ is not always sufficient to distinguish structural isomers—compounds with identical molecular formula but different chemical bond arrangements between atoms—and stereoisomers—compounds with identical formula and chemical bond arrangements but different spatial orientation of groups in the molecule [37,140,141]. In this case, additional separation methods (i.e., TIMS) in conjunction with MS^n^ may be required to validate the identification of a metabolite or lipid species. Of note, NMR represents a quicker and cheaper alternative (in terms of cost per sample) to MS^n^ with regard to structural elucidation [142].

### 4.2. Human Interindividual Variability and Potential Confounding Factors

**Figure 4:**
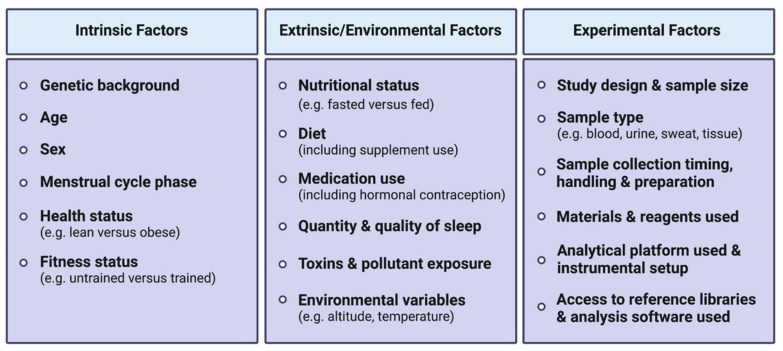
Summary of some of the main factors responsible for variance between metabolomics/lipidomics studies, including intrinsic, extrinsic/environmental factors and experimental factors.

One of the main challenges encountered in human exercise studies is the high interindividual variability in genetic background, sex, age, lifestyle, environmental exposure and nutritional and health status (Figure 4), which represent important confounding factors that are difficult to screen and control for in an experimental setting [10]. To overcome these challenges and account for the potential high interindividual variability amongst human participants, large-scale epidemiological studies are required [143]. Recruiting and analyzing such large numbers of individuals for a given experiment will be challenging (i.e., the appropriate sample size is variable depending on effect size, but hundreds of participants are often needed in human studies), as human exercise studies are usually performed using only small sample sizes (i.e., often less than one hundred). It should also be noted that overcoming high interindividual variability may be possible in small study groups through meticulous control of the above-mentioned confounding factors although this may lead to increased cost, time and constraints. Parallel exercise interventions using animal model systems is a complementary approach in which both genetic background and environment can be controlled to a greater extent compared to human cohorts.

Human metabolomics studies to investigate the molecular mechanisms of acute exercise are however starting to be performed at a larger scale. Indeed, a recent study investigated blood metabolic profiles of over 400 middle-aged adults, uncovering metabolic signatures associated with cardiometabolic health [144]. In addition, an ongoing initiative in the United States called The Molecular Transducers of Physical Activity Consortium (MoTrPAC) will address some of these remaining challenges in the decade ahead by examining the effects of acute and chronic exercise (including both endurance and resistance exercise) across a wide range of biological systems. This multi-site MoTrPAC initiative aims to analyze a large number of samples across pediatric, sedentary and highly active adult male and female human populations and complementary animal models using multi-omic approaches (including metabolomics/lipidomics), eventually establishing a comprehensive molecular map of exercise that will be made publicly available through the MoTrPAC Data Hub: https://motrpac-data.org (accessed on 15 December 2020) [145,146]. 

Metabolomics/lipidomics studies in the fields of sport and exercise physiology to date have mostly been conducted using only male participants, as highlighted in a recent human exercise metabolomics review [147], with only a few recent studies investigating acute exercise metabolomic/lipidomic patterns in obese and insulin resistant women [80,148,149]. The impacts of sex and hormonal variations (i.e., menstrual cycle phases) in females on exercise-induced metabolomic and lipidomic responses are therefore poorly understood, and more studies in female participants are warranted to begin to decipher these differences. These studies should take into account and report the use of hormonal contraception (including type of hormonal contraception used) in addition to the menstrual cycle phase during which the exercise is performed. This reporting is important as substantial differences in metabolic patterns are observed depending on menstrual cycle phase [150]. Likewise, aging is also associated with alterations in exercise-induced metabolomic responses. Therefore, continued efforts to identify new exercise-regulated biomarkers associated with aging and age-related pathologies such as muscle loss in sarcopenia may help personalize exercise interventions to prevent, delay or treat these age-related disorders [9]. As highlighted in previous sections, sampling certain tissues such as liver, which are relatively inaccessible in human exercise studies, can be more readily obtained using animal model systems. Since exercise-induced adaptations do not just involve changes in circulating, muscle and liver metabolites/lipids, animal models also provide more access to less-studied tissues (e.g., heart, brain) involved in the whole-body molecular metabolic responses to exercise.

### 4.3. Comparison and Reproducibility of Results Between Studies

Another major challenge in exercise-related metabolomics and lipidomics studies is the ability to directly compare studies between independent studies and research groups. The current lack of reproducibility and the common discrepancies observed within a given research field may in part be attributed to intrinsic and extrinsic/environmental confounding factors described in the previous section, as well as experimental factors (see Figure 4). Study designs should report or control for these factors (e.g., reporting dietary intake and timing and/or providing standardized meals at set times). Included in these experimental factors is the use of a wide variety of analytical platforms and data acquisition modes. Indeed, each analytical platform and detection mode is associated with specific sample handling, metabolite extraction and data acquisition/processing protocols and requirements. Although representing a valuable means to broaden metabolite coverage, these differences in instrumentation and analytical workflows contribute to substantial inter-study discrepancies that make reproducibility and data comparison between independent research groups a challenging and tedious process. Despite the fact that instrumentation-induced variability between studies cannot likely be solved due to differences in equipment between research facilities, harmonization in sample handling and data acquisition/processing protocols, along with standardized metabolite reporting are necessary to help overcome some inter-study discrepancies. This will allow more confident inter-study dataset comparisons, and subsequently improved data interpretation and biological insights. In 2007, the MSI proposed a consensus regarding minimum reporting standards for metabolite identification [51]. Similarly, the LSI also provides guidelines for lipid species annotation [151,152]. However, efforts to enforce adequate use and constant updates by the metabolomics community are necessary since, up until recently, the use of these reporting standards allowing investigators to define the level of compound identification/annotation confidence was suggested to be relatively low [141].

### 4.4. Bioinformatic Resources

To deal with the complexity and heterogeneity of metabolomics and lipidomics datasets (e.g., wide concentration range suggested to be spread over 12 orders of magnitude [139]) and the large amount of data generated by untargeted approaches, robust computational and bioinformatics resources and expertise are required. This is critical for data processing, analysis, interpretation and visualization. Numerous open-source and commercial data processing tools are available, but the overall lack of uniformity among these tools can also hinder reproducibility of findings between independent studies and research groups. Each tool has its own characteristics, but comparison of the performances of different tools has rarely been performed. Although software packages such as XCMS Online, SIEVE™ and Compound Discoverer™ provide reproducible and consistent data processing results, they have shown differences in metabolite selection, for example as candidate biomarkers for Alzheimer’s Disease [153]. Therefore, variations in data analysis among these different software packages should be carefully considered, and ideally systematic comparison of all packages utilized in untargeted metabolomics/lipidomics should be performed to help maximize data confidence, consistency in data handling, and reliability and reproducibility of biological findings. Alternatively, utilizing multiple software packages for data handling and only considering overlapping compounds for subsequent analysis may help reduce false positive and false negative compounds in datasets [153]. In addition, data analysis code should be provided as open access, as lack of transparency and reporting standards has led to widespread concerns in the reproducibility and integrity of results. Metabolomics researchers are encouraged to share their resources to provide adequate evidence of reproducibility. Collaborative cloud computing and Jupyter Notebooks are becoming popular amongst many metabolomics research groups and seem to be favored, as they provide added flexibility when compared to many of the online data repositories [154]. Metabolomics users are encouraged to use open-source platforms and adopt the FAIR data principles (Findable, Accessible, Interoperable, and Reusable) [155], promoting the use of open data formats, online spectral libraries and data reproducibility. 

## 5. Future Directions and Potential Value for Human Performance and Exercise Metabolic Health Benefits

Untargeted metabolomics/lipidomics is a hypothesis generating method and as such, future work should also focus on following up on these generated hypotheses, notably by using targeted approaches to validate findings and provide more quantitative insight (i.e., absolute metabolite/lipid concentrations) into exercise-regulated metabolites/lipids and biochemical pathways. Although metabolite and lipid concentrations provided by these targeted approaches are crucial to enhancing the measurement accuracy of exercise-induced metabolite changes, they alone only provide a snapshot of metabolic reactions that have just occurred. Therefore, complementary analyses such as metabolic flux analysis, also called fluxomics—a method that combines stable isotope tracing of metabolites with MS or NMR spectroscopy—help depict metabolic reaction capacities, therefore allowing more mechanistic insight into the dynamics of molecular metabolic reactions that may explain, at least partially, observed differences in metabolite concentrations between and/or within individuals over a certain time or exercise intervention [156]. In addition, the implementation of more multi-omics approaches in addition to metabolomics/lipidomics will enable researchers to gain deeper understanding of the complexity and interconnected nature of genetic, epigenetic, transcriptional, protein and post-translational networks underlying metabolomic responses to exercise [9]. Finally, repeat sampling (i.e., longitudinal tracking) is required to understand individuals at the systems level, and systems biology approaches will help revolutionize the study of exercise physiology. Moving from the traditional reactive approach, where an individual may experience fatigue through overtraining, or fail to respond to a bout of exercise conditioning, the emergence of systems biology will provide the ability to predict when an occurrence will occur. This in turn will facilitate more personalized medicine. Personalized medicine or P4 medicine (Predictive, Preventive, Personalized and Participatory) can provide new approaches for: developing personalized treatment strategies [157] that may include personalized training interventions; deepen our understanding of physiological processes; and ultimately expand our knowledge of the health continuum.

With respect to compound identification, the numerous and constantly evolving technologies in the metabolomics and lipidomics fields also provide tremendous potential for metabolomics and exercise research communities in the next decade. For example, the combination of IMS with chromatography and MS methods will help increase metabolite identification capacity. IMS-MS instruments are now available and implementation of IMS to current analytical platforms used in the field of exercise will help improve coverage of identified metabolites, therefore expanding capacity to map molecular responses to exercise. Likewise, the implementation of multidimensional approaches—where several separation methods are combined into a single analysis [37] to enhance compound separation and structure characterization—are expected to lead to substantial improvements in compound identification capacity in the next decade. 

Advancement in technologies will also aid how omics research of human exercise and athletic performance will be taken from the “bench and into the community.” Considerable interest in the use of dried blood spots (DBS and miniaturization technologies) in human performance fields may offer several important advantages over conventional whole blood sample collection. First, sample collection is less invasive compared to venepuncture and easy to perform (e.g., finger prick for adults, and heel prick for infants). This is particularly important for maximizing participant recruitment in the context of frequent repeated blood sampling. Second, blood sampling can be performed by an individual and away from a laboratory setting, such as the home of a clinical patient following only minimal training. Third, the use of miniaturized devices allows for low volume of blood to be collected (typically 10–200 μL), compared to standard venepuncture sampling which requires a minimum of ~2 mL of whole blood. Finally, as the name implies, samples are air-dried and can be shipped by mail to laboratories at minimal expense and without the need for maintaining stable temperature environments. Such devices will benefit from discovery research, where stable and quantifiable metabolites can be selected and developed for measurement of metabolites collected via miniaturized technologies. Considerable work is already occurring with DBS and dried urine spots, and companies are now developing technologies which can provide users with live measurement of metabolite concentrations which might be useful indicators of health or fitness phenotypes. These approaches will facilitate personalized exercise interventions and provide sport and exercise scientists with data that can inform decision making, health tracking and athlete phenotypes. 

In other mammalian species, recent efforts to utilize mouse genetic diversity panels and recombinant inbred mouse strains in metabolomics/lipidomics research have proven valuable tools for genetic mapping (i.e., quantitative trait loci mapping) and investigating environmental exposure, providing new insights into compound identification, as well as the molecular basis of metabolic health and disease. For example, a recent large-scale genome-lipid associated map and resource termed LipidGenie was generated by analysing liver and plasma samples from diversity outbred mice, which permitted the identification of unknown lipids from MS data by mapping molecular lipid features to genetic loci [158]. In addition, recombinant inbred mouse strains (e.g., ILSXISS) have been used to determine skeletal muscle metabolomic signatures reflective of IR across different mouse strains and diets [159]. Such determinations of molecular metabolite/lipid classifiers and predictors of metabolic disease-related phenotypes can be leveraged in future studies to help predict metabolic health status and potentially determine how an organisms’ metabolism may benefit from specific diets and/or exercise interventions.

In the next decade the exercise biology and metabolism fields will continue to benefit from these ongoing efforts in the metabolomics and lipidomics research community. Metabolomics/lipidomic analyses may ultimately be capable of being performed as part of routine health checks to assess an individual’s metabolic status (e.g., nutritional state, training state, pathology) and responses to a given stimuli (e.g., food intake, exercise, drug treatment) as well as predisposition to certain diseases. This will in turn enable highly tailored and personalized exercise, diet, and/or medical interventions to prevent, delay and combat metabolic disease [160].

## 6. Conclusions

The unbiased global measurement of metabolomes and lipidomes from different sources such as biological fluids and tissues are constantly improving. They represent a promising avenue to unravel the complex and interconnected metabolite and lipid networks underlying the molecular responses to exercise throughout the body. In the context of exercise biology, the application of metabolomics and lipidomics as a hypothesis-generating approach has dramatically increased the number of biological targets measured simultaneously thanks to the major technological advances over the last decade. This has allowed the measurement of hundreds to thousands of metabolites (including lipid species) in a single run and subsequently revealed biomarkers of exercise intensity, training state and exercise capacity, fatigue, among others. In summary, findings made over the past decade have revealed numerous metabolites regulated by acute aerobic/endurance and resistance exercise in various biofluids and tissues across mammalian species, including: glycolysis end-products and TCA cycle intermediates; FA, acylcarnitines and TAGs; ketone bodies; nucleotides and derivatives; amino acids and derivatives; vitamins; steroid hormones; as well as less characterized complex lipids with various functions, pro- and anti-inflammatory properties and vasoactive properties (Figure 3).

Although considered the closest reflection of an organism’s metabolic phenotype, the metabolome and the lipidome alone will not be sufficient to understand to complexity of exercise’s molecular landscape. Therefore, extensive efforts to integrate metabolomics and lipidomics data with other layers of biological regulation such as the genome, proteome and phosphoproteome will also be required. Importantly, expansion of metabolite and lipid databases will convert a much higher proportion of data into useful information and meaningful biological insights. In addition, continued advancements in instrumentation and analytical platforms in the metabolomics and lipidomics field will help standardize and harmonize experimental procedures from study design, sample handling, data acquisition, processing and analysis, as well as reporting. Together these efforts in the next decade will help maximize the utility of metabolomic and lipidomic profiling in exercise biology, metabolic health and disease, and beyond.

## Supplementary Materials

The following are available online at https://www.mdpi.com/2218-1989/11/3/151/s1, Figure S1: Lipid mediators derived from polyunsaturated fatty acids (PUFA), Table S1: Summary of discussed acute exercise studies in humans and other mammals using metabolomic approaches, Table S2: Summary of discussed acute exercise studies in humans and other mammals using lipidomic approaches.
