# Supplementary material for: Metabolomics and Lipidomics: Expanding the Molecular Landscape of Exercise Biology
Source: Metabolites. 2021 Mar 7;11(3):151. doi: 10.3390/metabo11030151 (PMC8001908; doi:10.3390/metabo11030151)
Supplement: Supplementary file 1 [file metabolites-11-00151-s001.pdf]

metabolites

MDPI

Supplementary Materials

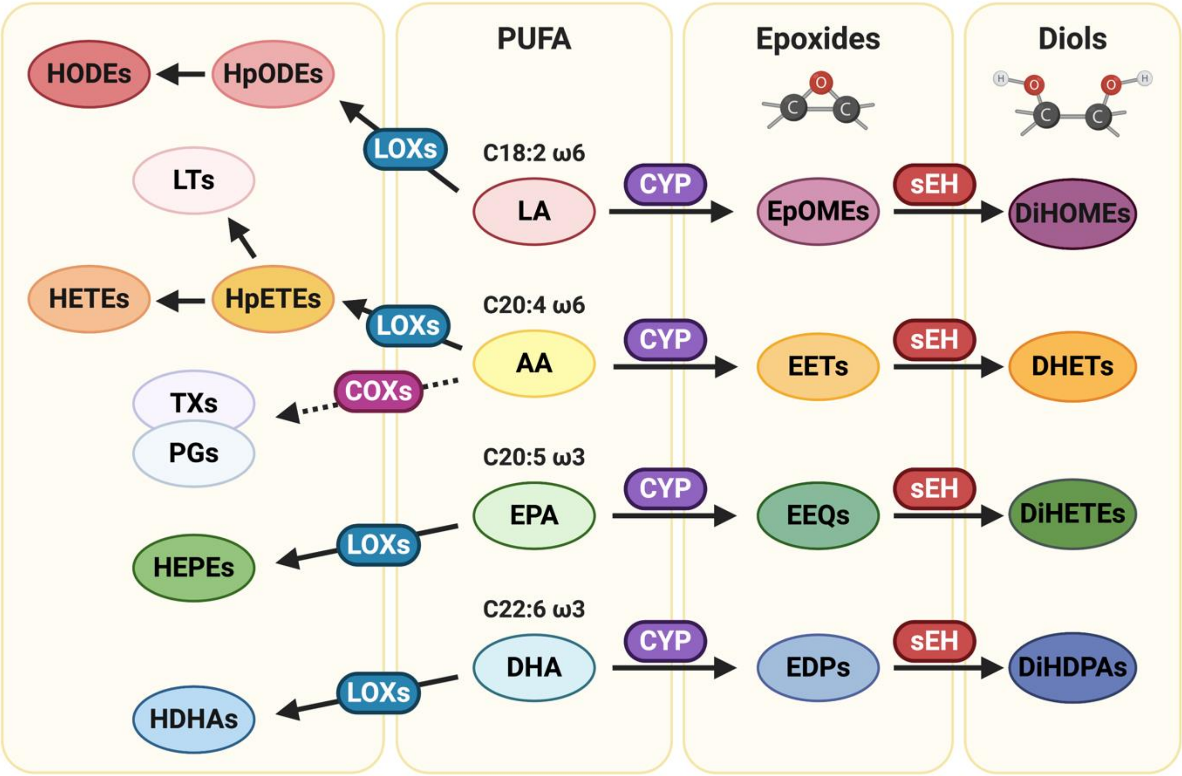

Figure S1. Lipid mediators derived from polyunsaturated fatty acids (PUFA). Depending on the intervening enzymes, different lipid mediators can be generated from a given PUFA. Cytochrome P450 (CYP) mono-oxygenase converts PUFA (LA: linoleic acid; AA: arachidonic acid; EPA: eicosapentaenoic acid; and DHA: docosahexaenoic acid) to their respective epoxides (EpOMEs: epoxyoctadecenoic acids; EETs: epoxyeicosatrienoic acids; EEQs: epoxyeicosatetraenoic acids; EDPs: epoxydocosapentaenoic acids) which exist in different regioisomers. These epoxide isomers are in turn converted to their respective diols (DiHOMEs: dihydroxyctadecenoic acids; DHETs: dihydroxyeicosatrienoic acids; DiHETEs: dihydroxyeicosatetraenoic acids; DiHDPAs: dihydroxydocosapentaenoic acids) under the action of the soluble epoxide hydrolase (sEH). Conversely, PUFA can also be metabolized by lypoxygenases (LOXs) which comprise three isoforms (5-, 12- and 15-LOX). LOXs are responsible for generating hydroxyperoxy-octadecadienoic acids (HpODEs), further metabolized to hydroxy-octadecadienoic acids (HODEs), from LA. If the substrate is EPA or DHA, hydroxyEPAs (HEPEs) and hydroxyDHAs (HDHAs) will be respectively generated via LOXs. From AA, LOXs can also produce hydroperoxyeicosatetraenoic acids (HpETEs), subsequently converted to either hydroxyeicosatetraenoic acids (HETEs) or leukotrienes (LTs) from AA. Finally, COX enzymes, which exist in two isoforms (COX-1 and COX-2) are responsible for the production of prostaglandins (PGs) and thromboxanes (TXs) from AA. Dotted arrow indicates indirect reaction.

Table S1. Summary of discussed acute exercise studies in humans and other mammals using metabolomic approaches

Authors & Year	Study Population	Exercise Characteristics	Analytical Platform; Biological Specimen	Study Design	Key Findings
Schranner et al., 2020 [62]	27 human studies (men and women, various ages, body compositions and fitness levels)	Various types, intensities and durations	Various platforms including NMR spectroscopy, LC-MS and GC-MS (targeted and untargeted); Blood plasma and serum, urine, sweat	Systematic review following PRISMA guidelines. Search for human metabolomics studies that report metabolite concentrations before and within 24 h after endurance or resistance exercise	**196 significantly changed metabolites within 24 h after exercise in ≥ 2 out of 57 experiments: 95 lipid species, 53 amino acids and derivatives, 14 nucleotides, 13 carbohydrates, 7 TCA cycle intermediates, 6 vitamins and co-factors, 5 xenometabolites and 3 peptides**<br>- ↑ lactate, pyruvate, TCA cycle intermediates<br>- ↑ 37 FA, 17 acylcarnitines, and ketone bodies (endurance)<br>- ↓ bile acids and several membrane lipids (endurance)<br>- Mixed responses in proteinogenic and non-proteinogenic amino acids
Lewis et al., 2010 [63]	25 amateur runners (aged 42 ± 9 years, men & women)	Moderate to high intensity, long duration: Marathon running (247 ± 46 min)	Targeted LC-MS; Blood plasma	Boston Marathon. Repeated measures from one cohort, blood collection before and within 10 min post-race	**88 metabolites quantified**<br>- ↑ products of adenine nucleotides catabolism: AMP, inosine, hypoxanthine, xanthine<br>- ↑ lipolysis (glycerol) and ketogenesis (β-hydroxybutyrate)<br>- ↑ glycolysis products (glucose-6-phosphate, 3-phosphoglycerate, pyruvate, lactate) and TCA cycle intermediates<br>- ↑ tryptophan metabolites: kynurenate, quinolinate, anthranilate<br>- ↑ niacinamide (insulin sensitivity modulator)<br>- ↓ gluconeogenic amino acids: alanine, threonine, serine, proline, valine, histidine, glutamine, asparagine
Stander et al., 2018 [64]	31 amateur runners (aged 41 ± 12 years, 19 men & 12 women)	Moderate to high intensity, long duration: Marathon running (259 ± 49 min)	Untargeted 2D GC-MS; Blood serum	Druridge Bay Marathon. Repeated measures from one cohort, blood collection the day preceding the race (≥2h fasted) and immediately post-race	**70 metabolites identified**<br>- ↑ glycolysis products (pyruvate), gluconeogenesis metabolites (e.g. myo-inositol, glycerol, glyceric acid) and TCA cycle intermediates<br>- ↑ FAs, OCFAs, alpha-hydroxyacids, and ketone bodies<br>- ↑ oxidative stress, ↑ cholesterol catabolism<br>- ↓ amino acids
Shi et al., 2020 [70]	20 amateur runners (aged 29 ± 5 years, all men)	Moderate to high intensity, long duration: Marathon running (average 160 min, all within 180 min)	Untargeted LC-MS; Blood serum	Shanghai International Marathon. Repeated measures from one cohort, blood collection 24h pre-race (fasted) and within 60 min post-race	**31 significantly changed metabolites between pre- and post-race**<br>- ↑ glycolysis products (pyruvate), gluconeogenesis metabolites (glycerol, glyceric acid) and TCA cycle intermediates<br>- ↑ lipolysis products: glycerol, glyceric acid, octanoic acid, quinic acid<br>- ↑ urea and ↓ glucosamine<br>- ↑ caffeine metabolism: xanthine, theophylline, theobromine<br>- ↑ cortisol and ↓ testosterone<br>- ↓ amino acids (including gluconeogenic): valine, serine, asparagine but ↑ alanine, tyrosine, phenylalanine

Table S1. *Cont.*

Authors & Year	Study Population	Exercise Characteristics	Analytical Platform; Biological Specimen	Study Design	Key Findings
Schader et al., 2020 [73]	76 amateur runners (aged 43 ± 11 years, all men)	Moderate to high intensity, long duration: Marathon running (225 ± 43 min)	Targeted LC-MS & FIA-MS; Blood plasma	Munich Marathon. Repeated measures from 3 groups: low, average, top performers (based on VO_2max and race time). Blood collection at 5 time points: 5 weeks and 1 week pre-race, then immediately, 24h and 72h post-race (all fasted except for immediately post-race sample, although before post-race refeeding)	**188 metabolites quantified**<br>For all cohorts immediately post-race:<br>- ↑ fat metabolism: acylcarnitines<br>- ↑ proxy for CPT enzymes activity: ratio palmitoylcarnitine + stearoylcarnitine/free carnitine<br>- ↑ arginine related metabolites and ↓ most amino acids<br>↓ phospholipids<br>Low vs top performers:<br>- Post-race acylcarnitines in low performers > top performers<br>- Post-race arginine-related metabolites in low performers > top performers
Manaf et al., 2018 [77]	18 active young men (aged 25 ± 5 years)	Moderate intensity, long duration: Time-to-exhaustion cycling test at 3mmol/L lactate (80 ± 14 min)	Untargeted LC-MS; Blood plasma	Repeated measures: blood collection 30 min pre-exercise (after a ≥10h overnight fast), 10 min into cycling (reference point instead of pre-exercise), before fatigue, immediately after fatigue, and 20 min post-fatigue	**80 metabolites identified with 68 significantly changed over time**<br>- ↑ lipolysis markers and fat metabolism: glycerol, carnitine, FA including oleic, palmitic acids and their acylcarnitines, acetylcarnitine<br>- ↓ tryptophan and ↑ serotonin (derived from tryptophan) end-product: 5-metoxy-3-indoleacetic acid<br>Changes in alternate tryptophan metabolism compounds: ↑ indole, indole-3-lactic acid, methyl indole-3-acetate; and ↓ indole-3-acetic acid<br>- ↑ neurotransmitters: gamma-aminobutyric acid, 2-aminobutyric acid<br>- ↑ dopamine degradation biomarker: homovanillic acid<br>- Mixed responses in amino acids: ↑ proline, ↓ citrulline, arginine, methionine; ↓ valine and glutamic acid with recovery; ↑ creatine
Contrepois et al., 2020 [27]	36 volunteers (aged 59 ±8 years, 58% men) with a wide range of insulin sensitivities	High intensity, short duration: Incremental treadmill run to exhaustion with a target duration of 8 to 12 min	Untargeted LC-MS; Blood plasma	Repeated measures; blood collection pre-exercise, then 2, 15, 30 and 60 minutes post-exercise (fasted). 15 participants provided a fasted blood sample the next morning for inter-day variability screening. A subset of the cohort (n=14) participated in a control trial (same blood collection protocol)	**728 metabolites identified, 4 clusters of longitudinal trajectories**<br>**Cluster 1: early ↑ post-exercise with quick return to basal levels**<br>- Glycolysis products (pyruvate, lactate); TCA cycle intermediates (malate); fat oxidation (FA and acylcarnitines); inflammation<br>**Cluster 2: delayed ↑ post-exercise with return to basal levels**<br>- TCA cycle intermediates (malate, citrate, a-ketoglutarate); adenine nucleotide catabolism (hypoxanthine and xanthine); uric acid; amino acids: alanine, tyrosine, glutamine, and proline<br>**Cluster 3: ↓ post-exercise with return to basal levels**<br>- Amino acids (glutamic acid, cystine, tryptophan, serine, threonine, and glycine); free carnitine; FA (C20-24)<br>**Cluster 4: ↓ post-exercise without return to basal levels**<br>- BCAAs (valine, leucine, isoleucine) and ↑ their degradation products (branched-chain ketoacids); caffeine metabolites; bile acids

Table S1. *Cont.*

Authors & Year	Study Population	Exercise Characteristics	Analytical Platform; Biological Specimen	Study Design	Key Findings
Hu et al., 2020 [81]	2 separate studies<br>A) 10 young healthy men (aged 23± 1 years)<br>B) 9 young healthy men (aged 21± 1 years)	Moderate intensity, long duration:<br>A) 2-hour cycling at 60% VO_2max<br>B) 2-hour continuous one-leg knee extension at 50% 1RM	Untargeted CE-MS and targeted LC-MS; Blood plasma	A) Repeated measures; plasma collected from hepato-splanchnic bed after an overnight fast before (at 0 min), during (at 60, 120 min) and after exercise (at 150, 180, 240, 300, 360 min) fasted.<br>B) Same plasma collection characteristics but from femoral artery (one leg) and femoral vein (both legs) up to 300 min post-exercise.<br>Note: liver transcriptomic data from male C57Bl/6N mice who ran 1h at 13m/min (14° incline) were also used [99]	**200 arterial plasma metabolites detected: 77 changed by exercise**<br>- ↑ medium- to long-chain FA during exercise and recovery, especially unsaturated FA<br>- ↑ ketone bodies only post-exercise<br>- ↑ TCA cycle intermediates (succinate, malate), lactate, hypoxanthine, FA 6:0, FA 7:0 and FA 8:0 during exercise with a ↓ during recovery<br>- ↓ most amino acids in the recovery phase<br>**Hepato-splanchnic flux of 21 metabolites changed during exercise**<br>- ↑ hepatic uptake of FA C6:0, C8:0, C14:0, C14:1, C16:1; TCA cycle intermediates (succinate, malate), glycolysis products (lactate and pyruvate), and amino acids arginine, glutamine and lysine<br>- ↑ release of saturated FA of ≥ C18; β-hydroxybutyrate, hippurate, aspartate, 3-methyl-2-oxovalerate and N5-ethylglutamine<br>**Metabolite flux from the working leg**<br>**-** ↑ uptake of β-hydroxybutyrate<br>- ↑ release of succinate, malate, FA C6:0 and C8:0<br>↑ **activation of HIF-, NRF2- and c-AMP-dependent transcription factors in mouse liver post-exercise**
Kistner et al., 2020 [82]	255 healthy volunteers (aged 46 ± 17 years, 148 men and 107 women)	High intensity, short duration:<br>Bicycle exercise to exhaustion (exercise tolerance test)	Targeted 1H NMR-spectroscopy; Urine	Repeated measures: spot urine collected (fasted) 90 min pre-exercise, then after a standardized breakfast and within 15-30 min post-exercise	**47 metabolites quantified: 37 significantly changed by exercise**<br>**-** ↑ lactate, acetate<br>- ↑ TCA cycle intermediates: citrate, cis-aconitate, succinate<br>- ↑ amino acids: glycine, histidine, isoleucine, leucine, taurine, threonine, tyrosine, valine<br>- ↑ BCAA degradation: methylsuccinate and 3-hydroxyisovalerate (leucine-derived metabolites), 3-aminoisobutyrate (valine-derived metabolite)<br>- ↑ carnitine and creatine
Harshman et al., 2018 [95]	11 active duty military volunteers (aged 24 to 42 years, 7 men and 4 women)	Low, moderate and high intensity, short to long duration:<br>Treadmill march with 22-kg gear (22 to 211 min)	Untargeted LC-MS; Sweat	Single sweat collection in the 3 subgroups following low (4.8 km/h, 3% incline, n=5), moderate (5.1 km/h, 4% incline, n=4) or high intensity (5.6 km/h, 6% incline, n=2) march in a control trial (same blood collection protocol)	**29 confirmed metabolites of 48 compounds tentatively identified**<br>**-** amino acids are the most abundant metabolites in sweat<br>- strong relationships between sweat metabolites (positive and negative)<br>- no significant metabolites associated with exercise duration or intensity

Table S1. *Cont.*

Authors & Year	Study Population	Exercise Characteristics	Analytical Platform; Biological Specimen	Study Design	Key Findings
Sato et al., 2019 [98]	C57Bl/6J mice (10-11 weeks of age, all male, n=6 per time point)	Low to moderate intensity, long duration: Treadmill running (60 min) at 6m/min with added 2m/min every 2 min up to 16m/min	Untargeted LC-MS & GC-MS; Skeletal muscle (*gastrocnemius* and quadriceps)	4 groups: exercise and control sedentary, both at early rest phase and early active phase. Measures at 6 time points in each group following exercise at 0, 4, 8, 12, 16 and 20 hours post-exercise	**672 metabolites detected (580 of known identity)**<br>Exercise in early active phase:<br>- ↓ temporal glucose metabolism<br>- ↑ amino acid breakdown, lipid oxidation, glycolysis, ketone metabolism<br>Exercise in early rest phase:<br>- ↑ temporal glucose metabolism<br>- ↓ temporal glycerol metabolism
Overmyer et al., 2015 [99]	HCR and LCR rats, both bred from N:NIH colony (4.5 months of age, all male, n=4-6/group)	High intensity, short and long duration: Incremental treadmill run to exhaustion (LCR: ~10 min; HCR: ~ 45 min)	Targeted LC-MS & GC-MS; Blood plasma and skeletal muscle (*gastrocnemius*)	2 groups: LCR and HCR. Measures at rest (0 min) and 10 min (immediately post-exhaustion) in LCR; and at 0, 10 and 45 min (immediately post-exhaustion) in HCR	**↓ fat uptake/oxidation and amino acid utilization during exercise in LCR vs HCR but similar mechanism of exhaustion (delayed in HCR)**<br>Plasma<br>- ↓ FA in LCR and HCR at 10 min<br>- ↓ TAGs in LCR < HCR at 10 min<br>- ↑ long-chain acylcarnitines in LCR at 10 min<br>- Small changes in medium-chain acylcarnitines in HCR at 10 min<br>- ↑ medium- and long-chain acylcarnitines in HCR at 45 min<br>- ↓ BCAAs and ornithine in HCR at 10 min<br>Skeletal muscle<br>- Small changes in long-chain acylcarnitines in LCR but ↑ in HCR at 10 min<br>- ↑ medium- and long-chain acylcarnitines in HCR at 45 min<br>- ↓ BCAAs and ornithine in LCR < HCR at 10 min
Klein et al., 2020 [100]	Standardbred horses (3-8 years of age, 4 male and 4 female)	High intensity (low to high), long duration: Incremental treadmill run to exhaustion (6% incline)	Untargeted LC-MS; Skeletal muscle[1] (*M. gluteus medius*)	All horses were used as both exercised and standing control in a crossover fashion. Repeated measures: 30 min pre-exercise, 3h and 24h post (food allowed after the 3h post-exercise biopsy). This protocol was repeated after a 12-week training followed by 72 hours of recovery	**545 metabolites identified**<br>Untrained state<br>- 31 metabolites changed 3h post (↑ 29; ↓ 2): ↑ 14 metabolites related to amino acid metabolism, other ↑ metabolites related to lipid (5), carbohydrate (3), nucleotide (3) vitamin/co-factor (1), and energy metabolism (1), 2 xenometabolites 3h post-exercise<br>- ↓ ribose 1-phosphate and fumarate (carbohydrate and energy metabolism)<br>- At 24h post-exercise, ↑ 1 metabolite (N-methylalanine)<br>Trained state<br>- ↑ 100; ↓ 42 metabolites 3h post. Metabolite changes were related to amino acid (↑ 37; ↓ 7), lipid (↑ 25; ↓ 24), xenometabolites (↑ 14), nucleotide (↑ 11; ↓ 2), carbohydrate (↑ 10; ↓ 2), and vitamin/co-factor (↑ 3; ↓ 1) metabolism<br>- ↑ 13 and ↓ 137 metabolites 24 h vs 3h post. Metabolite changes were related to ↓ in lipid, amino acid and nucleotide metabolism<br>- ↑ 74 other metabolites 24h post, 41 of 45 metabolites ↓ at 24 h vs 3 h in the untrained state also ↓ in the trained state

Table S1. *Cont.*

Authors & Year	Study Population	Exercise Characteristics	Analytical Platform; Biological Specimen	Study Design	Key Findings
Zhang et al., 2019 [101]	A) Sprague Dawley rats (adult males, n=9-10/group)<br>B) 5 healthy volunteers (aged 21-46 years, 3 men and 2 women)	Moderate intensity, short duration:<br>A) 20-min treadmill run at 18m/min (~60% VO_2max)<br>B) 30-min ergometer cycling at 65% heart rate max	Untargeted GC-MS;<br>A) Skeletal muscle (*gastrocnemius*) IF and blood plasma<br>B) Skeletal muscle (*v. lateralis*) IF	A) All rats used as resting controls and exercise treatment (crossover with one week of interval). Blood plasma and IF collected within 10 min post-exercise<br>B) Repeated measures, pre-exercise and during exercise (30 min into running)	**456 metabolites detected in muscle IF and plasma in rats (43% of all metabolites detected in both media, and 20% of exercise-changed metabolites present in both media)**<br><u>Rat muscle IF</u><br>**299 metabolites detected**<br>- ↑ most amino acids and derivatives; ↓ isoleucine, leucine, aminomalonate<br>- ↑ oleic acid, LA, AA, pelargonic acid, β-hydroxybutyrate ; ↓ lauric, palmitic, heptadecanoic, stearic, arachidic and lignoceric acids<br>- ↑ fructose, mannose, lactate, citric acid, alpha-ketoglutarate, oleamide, N-acetyl-D-hexosamine and ethanolamine; ↓ glucose-6-phosphate and 3-phosphoglycerate, nicotinic acid, glycerol-alpha-phosphate<br><u>Rat plasma</u><br>**353 metabolites detected**<br>- ↓ amino acids and urea; ↑ ketone: β-hydroxybutyrate<br>- ↑ unsaturated FAs: oleic and palmitoleic acids, LA and AA, and long-chain saturated FAs palmitic and stearic acids;<br>- ↑ fructose and lyxose; ↓ arabitol and lyxitol; ↑ cytosine and thymidine<br>- ↑ TCA cycle intermediates (citric acid, isocitric and aconitic acids and alpha-ketoglutarate)<br><u>Human muscle IF</u><br>**414 metabolites detected** (including 142 also detected in rat IF). Of the metabolites changed by exercise in rat IF, 78 detected in humans and 70% changed in the same direction
Huang et al., 2010 [102]	Sprague Dawley rats (6 weeks of age, all males, n=5/group)	High intensity, long duration:<br>Incremental run to exhaustion on 10% inclined treadmill at up to 30m/min	Untargeted GC-MS;<br>Liver	3 groups: sedentary control, exhaustive exercise and endurance training[2]. Livers collected in control group and immediately post exhaustive exercise	**55 metabolites identified**<br>- ↑ energy consumption markers: adenosine metabolites xanthine and hypoxanthine; creatinine<br>- ↓ carbohydrates and lactate<br>- ↑ FA and ketone bodies<br>- ↑ ornithine, urea<br>- ↑ pro-inflammatory precursors: AA, LA, oleic acid

1H NMR: proton nuclear magnetic resonance; AA: arachidonic acid; BCAA: branched-chain amino acid; CE: capillary electrophoresis; CPT: carnitine palmitoyl transferase; DAG: diacylglycerol; FA: fatty acids; FIA: flow-injection analysis ; GC: gas chromatography; HCR: high running capacity; IF: interstitial fluid; LA: linoleic acid; LC: liquid chromatography; LCR: low running capacity; MS: mass spectrometry; OCFA: odd-chain fatty acid; TAG: triacylglycerol; TCA: tricarboxylic acid.

[1] Plasma BCAA concentrations were also assessed in this study but were not discussed in this review

[2] Results from the endurance training protocol are not described since this review is focused on acute exercise only

Table S2. Summary of discussed acute exercise studies in humans and other mammals using lipidomic approaches

Authors & Year	Study Population	Exercise Characteristics	Analytical Platform; Biological Specimen	Study Design	Key Findings
Gollasch et al., 2019 [106]	6 healthy volunteers (aged 38 ± 15 years, 1 woman & 5 men)	High intensity, short duration: Incremental treadmill run to exhaustion	Targeted LC-MS; Blood plasma	Bruce protocol [108]. Repeated measures; blood collection pre-exercise, during (heart rate at 150 beats per min), immediately and 10 min post-exercise	**Post-run (immediately and/or 10 min post):**<br>- ↑ LA-derived epoxide: 12,13-EpOME<br>- ↑ AA-derived diol: 5,6-DHET<br>- ↑ EPA-derived diols: 5,6- DiHETE and 17,18-DiHETE<br>- No effect on the majority of CYP and LOX lipid metabolites
Stanford et al., 2018 [112]	Cohort 1:<br>27 healthy young and older men with various physical activity levels<br>Cohort 2:<br>12 healthy young adults (29 ± 1 years, 6 men & 6 women	Moderate intensity, short duration:<br>Cohort 1:<br>40 min cycling at 70% of heart rate reserve<br>Cohort 2:<br>45 min treadmill running at 75% of VO_2max	Targeted LC-MS; Blood plasma	Cohort 1: Repeated measures; blood collection following an overnight fast pre-exercise, immediately and 3h post-cycling<br>Cohort 2: Repeated measures; blood collection following an overnight fast pre-exercise, 15 min into, immediately and 1h post-run	Cohort 1:<br>**88 lipid mediators quantified**<br>- ↑ LA-derived diol: 12,13-DiHOME immediately post-exercise<br>- ↓ 13 lipids immediately post-run, including:<br>LA-derived mediators: 9- and 13-oxoODE; 13-HODE; epoxide 9,10-EpOME<br>- AA-derived mediators: epoxide 14,15-EET, 12-oxo-ETE, and LTB4 degradation product 12-oxo-LTB<br>- EPA-derived mediator: 9-HEPE<br>- DHA-derived mediator: 8-HDHA<br>Cohort 2:<br>- ↑ LA-derived diol: 12,13-DiHOME immediately post-exercise<br>From both cohorts:<br>- 12,13-DiHOME levels in active > sedentary participants before exercise<br>- ↑ 12,13-DiHOME in active > sedentary participants immediately post-exercise
Nieman et al., 2014 [115]	19 trained male cyclists (aged 38 ± 2 years)	Moderate intensity, long duration:<br>75-km cycling time trial (162 ± 4 min, mean intensity at 69±2 % of VO_2max)	Untargeted GC-MS & LC-MS;<br>Blood plasma	Repeated measures; blood collection following an overnight fast pre-exercise, immediately, 1.5h and 21h post-cycling	**Immediately and 1.5h post-cycling:**<br>**-** ↑ LA, LNA, dihomo-LNA, AA, DPA, adrenate<br>- ↑ LA-derived mediators: 9- & 13-HODE; 9,10-DiHOME; 12,13-DiHOME<br>- ↑ oxidative stress biomarker: F2-isoprostane<br>- Correlation of post-cycling 9- & 13-HODE with post-cycling AA; 12,13-DiHOME; F2-isoprostane, LA, dihomo-LA, adrenate
Gollasch et al., 2019 [117]	6 healthy volunteers (aged 38 ± 15 years, 1 woman & 5 men)	High intensity, short duration: Incremental treadmill run to exhaustion	Targeted LC-MS; Red blood cells	Bruce protocol [108]. Repeated measures; blood collection pre-exercise, during (heart rate at 150 beats per min), immediately and 10 min post-exercise	**Immediately and 10 min post-run:**<br>- ↑ LA-derived epoxides: 9,10- and 12,13-EpOME<br>- ↑ AA-derived epoxides: 5,6-EET, 11,12-EET, 14,15-EET<br>- ↑ DHA-derived epoxides: 16,17-EDP and 19,20-EDP<br>- No significant effect on diols, LOX and COX lipid mediators
Gollasch et al., 2019 [119]	6 healthy volunteers (aged 38 ± 15 years, 1 woman & 5 men)	High intensity, short duration: Incremental treadmill run to exhaustion	Targeted LC-MS; Red blood cells & blood plasma	Bruce protocol [108]. Repeated measures; blood collection pre-exercise, during (heart rate at 150 beats per min), immediately and 10 min post-exercise	**20 FAs quantified in RBCs and plasma (C12:0 to C22:6):**<br>- No effects of exercise on RBC FA levels, including omega-3 quotient<br>- No changes in plasma FA following exercise<br>- ↓ RBC lauric acid (C12:0) between exhaustion and 10 min post-exercise

Table S2. *Cont.*

Authors & Year	Study Population	Exercise Characteristics	Analytical Platform; Biological Specimen	Study Design	Key Findings
Contrepois et al., 2020 [27]	36 volunteers (aged 59 ±8 years, 58% men) with a wide range of insulin sensitivities	High intensity, short duration: Incremental treadmill run to exhaustion with a target duration of 8 to 12 min following warm-up	Semi-targeted LC-MS; Blood plasma	Repeated measures; blood collection pre-exercise, then 2, 15, 30 and 60 minutes post-exercise (fasted). 15 participants provided a fasted blood sample the next morning for inter-day variability screening. A subset of the cohort (n=14) participated in a control trial (same blood collection protocol)	**710 lipid species, 4 clusters of longitudinal trajectories**<br>**Cluster 1: early ↑ post-exercise with quick return to basal levels**<br>- Cholesteryl esters (n = 20), phosphatidylcholines (n = 23), DAGs (n = 10), ceramides (n = 9), and sphingomyelins (n = 8); long-chain unsaturated TAGs (including AA,EPA,DHA) markers of pro- and anti-inflammatory processes along with sphingolipids and ceramides<br>**Cluster 4: ↓ post-exercise without return to basal levels**<br>- Most TAGs (indicating lipolysis) especially saturated (indicating preferential use for energy conversion)
Vella et al., 2019 [124]	12 young active men (aged 22 ± 1 years)	High intensity, short duration: Maximal concentric and eccentric unilateral knee extension 3x12 repetitions at 60°/s	Targeted LC-MS; Skeletal muscle (*v. lateralis*)	Repeated measures; muscle biopsies pre-exercise following an overnight fast, then 2,4 and 24h post-exercise	**84 lipid mediators detected and quantified in the resting skeletal muscle**<br>All changes at 2h post-exercise back to basal at 4 and 24h post-exercise<br><u>COX pathway:</u><br>- ↑ AA-derived TXA2 biosynthesis markers : TXB2 and 12(S) HHTrE<br>- ↑ AA-derived PGE2 and PGF2α<br>- ↑ EPA-derived 15-DeoxyΔ12,14-prostaglandin J3<br><u>LOX pathway:</u><br>- ↑ AA-derived 5-HETE; 12-HETE + degradation product tetranor 12-HETE<br>- ↑ AA-derived LTB4 (undetectable at rest) and degradation products: 12-oxo-LTB and 20-COOH-LTB4<br>- ↑ EPA-derived 12-HEPE<br>- ↑ DHA-derived 7- and 14-HDHA<br><u>CYP pathway:</u><br>- ↑ AA-derived 5,6-EET; AA-derived 11,12- and 14,15-DHET<br>- ↑ LA-derived 9,10- and 12,13-DiHOME
Rivas et al., 2012 [125]	9 healthy young men (aged 22 ± 1 years) and 10 healthy older men (aged 74 ± 2 years)	High intensity, short duration: Knee extension & leg press: 3x10 repetitions at 80% of 1-RM	Targeted LC-MS; Skeletal muscle (*v. lateralis*)	Lipidomic analysis only pre-exercise muscle biopsies following an overnight fast and standardized meals (ad-libitum) the preceding 24h (muscle biopsies immediately and 6h post-exercise (fasted) for other analyses)	**Quantification of 9 intramuscular ceramides (C14:0 to C24:1)**<br>- No significant changes in total or unsaturated ceramides between older and younger men<br>- Palmitic (C16:0) and arachidic (C20:0) ceramides in young men < older men

Table S2. *Cont.*

Authors & Year	Study Population	Exercise Characteristics	Analytical Platform; Biological Specimen	Study Design	Key Findings
Markworth et al., 2013 [122]	16 healthy young men (aged 23 ± 1 years both control (n=8) and Ibuprofen (n=8) group)	High intensity, short duration:<br>10-min warmup followed by bar squats, leg press and knee extension. Circuit with 1-min inter-set rest and 3-min rest between different exercises<br>3x8-10 repetitions at 80% of 1-RM	Targeted LC-MS; Blood serum	Repeated measures; blood collection 15 min pre-exercise following an overnight fast (10h); every 30 min from 0 to 3 hours post-exercise; then 24 hours post-exercise	**87 lipid species detected and quantified**<br>- ↑ of 29 post-exercise (especially from 1 to 3h post-exercise)<br>- Most of lipid species were back to basal at 24h post-exercise<br><u>COX pathway:</u><br>- ↑ AA-derived TXA2 biosynthesis markers : TXB2 and 12(S) HHTrE<br>- ↑AA-derived PGE2 and PGD2, 15-keto PGE2 and 13,14-dihydro-15-keto PG, 15-keto PGF2α , 6-keto PGF1α<br>- ↑ EPA-derived RvE1<br><u>LOX pathway:</u><br>- ↑ AA-derived 12-HETE + degradation product tetranor 12-HETE<br>- ↑ AA-derived 15-HETE, 15-oxo-ETE<br>- ↑ AA-derived LTB4 , LXA4 and LXB4<br>- ↑ LA-derived 9- and 13-HODE, 9- and 13-oxo-ODE<br>- ↑ DHA-derived 10(S),17(S)-DiHDHA (protectin D1)<br><u>CYP pathway:</u><br>- ↑ AA-derived 11,12- and 14,15-DHET<br>- ↑ LA-derived 9,10- EpOME and 9,10-DiHOME
Nolazco Sassot et al., 2019 [128]	4 Thoroughbred horses (aged from 3 to 6 years, 3 male and 1 female)	High intensity, short duration:<br>Supramaximal treadmill run (115% VO_2max) to exhaustion	Untargeted LC-MS; Blood plasma	Repeated measures; blood collection pre-exercise following a 4-hour fast; immediately, 15 and 30 min post-exercise	**933 detected lipid species including 130 known lipids:**<br>**13 lipid species changed by supramaximal exercise:**<br>- ↑ 3 phosphatidylcholines: PC (p-34:1), PC (P-36:2) and PC (P-36:4)<br>- ↑ LPC (18:0)<br>- ↑ 2 sphingolipids: SM (d36:1) and SM (d42:2)<br>- ↑ 3 unsaturated FAs: LA (C18:2), LNA (C18:3) and 11,14-eicosadienoic acid (C20:2)<br>- ↓ 4 saturated FAs: C12:0, C14:0, C17:0 and C20:0
Hu et al., 2010 [132]	C57Bl/6J mice (all male, aged 12-weeks)<br>exercise group: n=8 sedentary group: n=8	Moderate intensity long duration:<br>60 min of treadmill running at 14 m/min and 14°incline after a 5-min warmup at 5m/min and 5° incline	Targeted LC-MS; Liver	Sedentary control group (mice stayed in their cage) and exercise group: liver collection (fed state) immediately and 3 hours post-exercise (with free access to food in the first 2 hours)	**115 lipid metabolites quantified**<br>- No significant differences in hepatic profile between rest and exercise immediately post-exercise<br>- 21 lipids mostly responsible (↑ 17 TAGs, ↓ 3 PCs and 1 LPC) for hepatic profile separation 3h post-exercise<br>- ↑ polyunsaturated TAGs (50:3; 54:5; 54:6; 54:7; 56:4; 58:6; and 58:10)<br>- ↓ PCs (36:1; 38:3; 40:4) and DAG (34:1)<br>- ↑ 63% in total liver TAG content in the recovery phase and ↓ content in skeletal muscle immediately post-exercise and in the recovery phase

Table S2. *Cont.*

Authors & Year	Study Population	Exercise Characteristics	Analytical Platform; Biological Specimen	Study Design	Key Findings
Hoene et al., 2016 [133]	C57Bl/6N mice (all male, aged 12-weeks, n=6) exercise and sedentary group	Moderate intensity, long duration: 60 min of treadmill running at 13 m/min and 13°incline after a 5-min warmup at 5m/min	Targeted LC-MS; Liver and skeletal muscle (*gastrocnemius* and *soleus*)	Exercised mice (fed state): liver, *gastrocnemius* and *soleus* muscle collection within 15 min post-exercise<br>Control sedentary mice (fed state): placed 60 min in new cages without food during trial)	**199 complex lipids and 33 FA (+ 39 acylcarnitines, acetylcarnitine and free carnitine) quantified in liver, gastrocnemius and soleus muscles at rest.**<br>**Pronounced profile differences between liver and skeletal muscle, but also between soleus and gastrocnemius:**<br>- Liver FA, ceramides, sphingomyelins and phospholipids > than in muscle - Soleus profile more similar to liver than gastrocnemius. Total carnitine, free carnitine, acetyl- and propionylcarnitine, short-chain hydroxylated acylcarnitines in soleus >> gastrocnemius. Medium- and long-chain acylcarnitines in soleus < gastrocnemius. Total and free carnitine levels in liver more similar to gastrocnemius than soleus<br>- Sum of short-chain acylcarnitines in liver >> soleus >> gastrocnemius<br>**Acute exercise changed the hepatic lipid profile**<br>- Most lipids not significantly changed by exercise in liver<br>- ↑ liver total lysophospholipids: LPCs (16:0; 18:2; 20:4; 22:5 and 22:6), LPEs (16:0; 18:0; 18:1 and 20:4)<br>- ↑ liver total plasmalogens: PE-P (P-38:4 and P-40:4)<br>- ↑ liver LPC/PC and LPE/PE<br>- ↑ free and total carnitine and ↓ acetylcarnitine (likely related to upregulation of mRNA of the carnitine transporter Slc22a5/OCTN2)<br>**Acute exercise changed the skeletal muscle lipid profile**<br>- Most lipids, free and total carnitine not significantly changed by exercise in skeletal muscles<br>- ↑ acetylcarnitine (soleus>gastrocnemius), hydroxy-acylcarnitines (soleus>gastrocnemius), and short-chain acylcarnitines in both muscles<br>**Palmitate flux differs between liver and skeletal muscles after exercise**<br>- Excess of circulating FA taken up by the liver and incorporated in TAGs and phospholipids during recovery

1-RM: one-repetition maximum; AA: arachidonic acid; COX: cyclooxygenase; CYP: cytochrome oxidase P450 ; DAG: diacylglycerol; DHA: docosahexaenoic acid; DHET: dihydroxyeicosatrienoic acid; DiHDHA: dihydroxyDHA; DiHETE: dihydroxyeicosatetraenoic acid; DiHOME: dihydroxyoctadecanoic acid; DPA: docosapentaenoic acid; EET: epoxyeicosatrienoic acid; EDP: epoxydocosapentaenoic acid; EPA: eicosapentaenoic acid; EpOME: epoxyoctadecenoic acid; FA: fatty acids; GC: gas chromatography; HDHA: hydroxyDHA; HEPE: hydroxyEPA; HETE: hydroxyeicosatetraenoic acid; HHTrE: hydroxyheptadecatrienoic acid; HODE: hydroxyoctadecadienoic acid; IR: insulin resistant; IS: insulin sensitive; LA: linoleic acid; LC: liquid chromatography; LNA: linolenic acid; LOX: lipoxygenase; LPC: lysophosphatidylcholine; LPE: lysophosphatidylethanolamine; LT (including B, B4): leukotriene; MS: mass spectrometry; oxo-ETE: oxo-eicosatetraenoic acid; oxo-ODE: oxo-octadecadienoic acid; PC: phosphatidylcholine; PE(-P): phosphoethanolamine; PG (including D2, E2, F1α and F2α): prostaglandin; RBC: red blood cell; Rv (including E1): resolvin; SM: sphingolipid; TAG: triacylglycerol; TX (including A2 and B2): thromboxane.
